# Supplementary material for: Persistence of Ebola virus in semen among Ebola virus disease survivors in Sierra Leone: A cohort study of frequency, duration, and risk factors
Source: PLoS Med. 2021 Feb 10;18(2):e1003273. doi: 10.1371/journal.pmed.1003273 (PMC7875361; doi:10.1371/journal.pmed.1003273)
Supplement: S1 Protocol — 25 January 2016. (DOC) [file pmed.1003273.s002.doc]

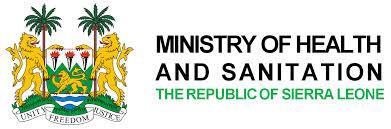


Study Protocol: Persistence of Ebola virus in body fluids of Ebola virus disease survivors in Sierra Leone

Version 12

11 December 2015


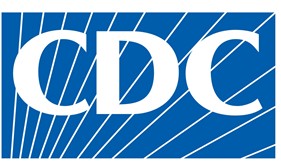

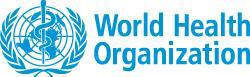

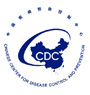


In collaboration with WHO, US-CDC and Chinese-CDC

**Table of Contents**

1.1 Background Information and Rational 6

1.2 Statement of the Problem and Objectives 7

1.2.1 Main objective 8

Sub-objective 8

1.2.2 Global framework of the study 8

1.2.3 Specific objectives of the Pilot study 8

1.2.4 Specific objectives of Main study 8

2. Study design 9

2.1.1 Study site selection 14

2.1.2 Study participants 15

A. Participant recruitment 15

B. Participant enrollment 16

Sample size calculations 16

2.2 Admission procedure 19

2.2.1 Follow-up procedures 21

2.2.2 Criteria for discontinuation of a participant 22

2.2.3 Laboratory and other investigations 22

3. Study instruments 24

4. Project management 24

4.1 Staffing and work plan 26

4.2 Administration and monitoring 26

5. Data quality assurance 27

6. Data management 27

7. Data analysis plan 28

8. Work plan and Study timeline 29

9. Main problems anticipated and proposed solutions 33

10. Applicability of results 33

11. Gender considerations 33

11.1 Describe how women and men are affected by the public health need that the study addresses, and whether this is a need expressed or felt by women and /or men 33

11.2 Explain how the research contributes to identifying and/or reducing inequities between

women and men in sexual and reproductive health and health care 33

11.3 Describe measures taken to facilitate the individual participation of women or men in the research process in light of their different life situations 33

11.4 Describe measures taken to ensure that community involvement is inclusive 34

11.5 Describe the sex composition of the research team, and their duties and responsibilities in the proposed research 34

12. Ethical considerations 34

12.1 Study population, recruitment strategy and informed consent process 34

12.2 Perceived risks and benefits of the study, both at the individual and community levels 34

12.3 Safeguards to protect any recognized vulnerability of the study participants 35

12.4 Reimbursement or compensation to study participants 35

12.5 Access to treatment or counselling for conditions either identified during screening of potential participants or resulting from the study intervention 35

12.6 Responsiveness of the project to community needs and priorities 35

12.7 Deception 35

**Project summary**

Little is known about the post-recovery persistence of Ebola virus (EBOV) and the risks of transmission from convalescent Ebola survivors during close or intimate contact. During this ongoing outbreak, it is important to fully understand how long the virus stays active in body fluids other than blood in order to reduce transmission and also which host factors determine persistence. Thus, we propose to investigate the persistence of the virus in different body fluids and its relation to immune response in a cohort of survivors, in order to target and refine public health interventions to arrest ongoing spread of disease. This will be an observational cohort study including survivors at different points of convalescence. Results and analyses will be used to update relevant counseling messages and recommendations from the Ministry of Health and Sanitation (MoHS), WHO, and US-CDC.

The study will be comprised of a pilot and a main study including specimens from adult men and women (serum, semen, vaginal secretions, and saliva, tears, sweat, urine, rectal swab, menstrual blood and breast milk if applicable), Participants will be recruited from Ebola treatment units (ETUs) and survivor registries and special efforts will be made to recruit HIV positive survivors. Participants in the study will be followed up at study sites in government hospitals.

Specimens will be tested for EBOV RNA by RT-PCR in Sierra Leone at 2-4 week intervals and the positive RT-PCR samples will be sent to US-CDC Atlanta for virus isolation. Each body fluid will be collected until two negative RT-PCR results at 2 weeks interval are obtained. Participants will be followed until all studied body fluids are negative. Analyses of antibody response, including titers of IgM and IgG, will be repeated in parallel to the body fluid tests.

Pilot and main study participants will be offered a follow-up visit with a questionnaire and body fluid testing at 3 and 6 months after their second negative RT-PCR test result.

Pregnant women will have an additional follow up to document the evolution of pregnancy, the outcomes of delivery, including products of delivery and body fluid testing. The process of this follow-up is detailed in the annex to this protocol

At each visit, specific prevention counselling and provision of condoms and infant formula (as applicable) will be executed by trained counsellors. Voluntary HIV testing and counseling will be offered to all participants and HIV positive study participants will be referred to national HIV services. Participants will be referred to survivor services for post-recovery complications as needed.

**1. Detailed description of the project**

**1.1 Background information and rationale**

The epidemic of Ebola virus disease (EVD) that began in 2014 in West Africa has been unprecedented in scale and duration. As of August 2, 2015, there have been over 27,862 reported cases and over 11,000 deaths, in the three most affected countries: Sierra Leone, Guinea, and Liberia. These case counts may underestimate the true case numbers and deaths due to underreporting. Recently, reported incidence has been falling in all three countries, but transmission and new cases continue to occur. As of August 2, 2015, a total of

7 cases had been reported in the previous 21 days in Sierra Leone, 27 in Guinea, and 0 in

Liberia. To date, the case fatality rate is estimated to be between 53 and 64% in the 3 countries. The precise number of survivors is unknown, but is expected to be in the thousands. Survivors are released from Ebola treatment units (ETUs) with a discharge certificate after two tests for EBOV in the blood have been negative by reverse transcription polymerase chain reaction (RT-PCR).

Anecdotal reports suggest that some new cases might be occurring from sexual transmission of the virus between EVD survivors and their sexual partners. In addition to concerns about semen and vaginal fluid, concerns exist regarding the unknown persistence of EBOV in other body fluids of survivors (e.g., saliva, sweat, tears, urine, rectal swabs, and breast milk), and their potential as a source of ongoing transmission; the literature does not rule out these possibilities.

After clinical improvement and elimination of viremia, limited data exist concerning EBOV clearance, persistence, and shedding during convalescence, including the relation to IgM and IgG response. It has been reported that EBOV can be detected in seminal fluids of a convalescent man at 82 days after onset of symptoms. Knowledge is scarce in relation to what influences Ebola persistence in body fluids following recovery from Ebola. It has been stipulated that sexual activity involving ejaculation may decrease viral load in semen, and likewise may influence results of test for detection. The putative association has been discussed in the literature, but has not been further examined so far.

Limited evidence suggests that live EBOV can persist in urine for 26 days following symptom onset. Evidence regarding EBOV in vaginal secretions is also limited. EBOV RNA has been detected in vaginal secretions at 33 days after symptom onset; due to the type of diagnostic test used (RT-PCR), it was not clear whether these traces represented live virus. Similarly, EBOV RNA was detected in sweat at 40 days after symptom onset, but no live virus was isolated. In addition, rectal swabs and conjunctival fluid have been found by RT-PCR to contain EBOV RNA at 29 and 22 days after illness onset, respectively. A limited number of saliva specimens have been tested by RT-PCR, all yielding negative results. Sexual transmission has never been definitively documented for EVD, although there have been a handful of anecdotal reports of suspected sexual transmission during the current outbreak.

The risk of sexual transmission of EBOV is not sufficiently documented. Prior to March 2015, guidance from World Health Organization (WHO) and The United States Centers for Disease Control and Prevention (US-CDC) recommend that Ebola survivors should abstain from sexual intercourse for 3 months (90 days) after the onset of symptoms, or use condoms if abstinence is not possible. Data which has emerged during this epidemic includes RT-PCR positive semen at 140 days post symptom onset and a case report of suspected sexual transmission in Liberia (March 2015) from a male survivor one of his female sexual partners (Christie et al., 2015; Mate et al., 2015). The male survivor had RT-PCR positive semen 199 days after symptom onset. Genetic sequencing of the viruses within this suspected transmission chain and limited epidemiological data suggested sexual transmission. Following the publication of this case report, as of 08 May 2015, both WHO and US-CDC updated their guidance on sexual practices of male EVD survivors. The WHO recommendations include abstinence or correct and consistent condom use for *at least* 6 months and for male survivors who have access to testing, to have their semen tested monthly from 3 months onwards; and for those who test positive, monthly thereafter until two consecutive RT-PCR negative results. Published RT-PCR data from the pilot phase of this study demonstrated RT-PCR positive semen samples 9 months after symptom onset (Deen et al., 2015).

Additionally, there is limited data on EBOV in breast milk. In one published case series, two mothers each provided a single breast milk specimen that was tested by virus isolation. One mother’s specimen had detectable virus at 7 days after disease onset and the second mother’s specimen had detectable virus at 15 days after disease onset. Testing began after EBOV was no longer present in either mother’s blood. Another study reported that three breastfed infants of mothers with EVD and one neonate with an unknown method of feeding died shortly after their mothers. In general, infants breastfeeding from women who had laboratory-confirmed EVD are considered to be at high risk of developing EVD themselves.

Given limited knowledge related to the presence and potential risk of transmission of EVD via breast milk, the current WHO and CDC recommendation is that both women with and recovering from EVD, who are caring for an infant who does not have EVD, should discontinue breastfeeding when safe replacements for breastfeeding and infant care exist. Another organization, UNICEF, recommends only that breastfeeding be discontinued for at least 8 weeks following recovery from EVD.

The implications for transmission, of viral persistence in body fluids outside semen and vaginal fluids as well as breast milk are unknown. Hypothetically body fluids containing virus may pose a risk of transmission during intimate contact such as between parent and young child, household contacts sharing beds, partners engaging in intimate but not penetrative sexual activity and in addition to health care staff caring for survivors in different functions. Thus far, epidemiologic data from the current West African epidemic does not point to intimate contact without exposure to semen with Ebola survivors after discharge from an Ebola Treatment Unit as being a risk factor for developing EVD.

The potential public health impact of EBOV transmission by contact with body fluids from convalescent survivors is substantial. Given the large number of survivors from the current outbreak in West Africa, even a small percentage of survivors who transmit EBOV to their sex partners, children, or other contacts could prolong clusters of infection or spread EBOV to new communities.

More recently a Scottish nurse experienced a severe complication with meningitis where Ebola virus detected in the cerebrospinal fluid and blood approximately 10 months after recovering from the initial infection. This raised questions on the frequency of this event among the population of survivors, and whether virus persisting in the body could be detected intemittently. These questions have to be better documented for the population of Ebola survivors in the three countries.

Many questions remain on how to manage pregnancy and delivery in Ebola survivors. Pregnant women who develop EVD have many complications, which include high mortality and stillbirths. In a few cases, a miscarriage occurred after a woman had resolved EVD infection in the blood, and the fetus and amniotic fluid was found to have virus. Little is known about whether virus can persist in women who have recovered from EVD and became pregnant after recovery.

*Ebola survivors in Sierra Leone*

The current situation for Ebola survivors in Sierra Leone involves several survivors’ support groups, actively working to provide risk reductioncounseling and support of various qualities to survivors.

In addition, survivors’ clinics are available, which provide medical care for post-Ebola sequelae, together with counseling, including around sexual transmission. The survivors are provided job opportunities in different organizations for community engagement to educate on stigma of sexual transmission; social mobilization and contact tracing. These activities are run in collaboration with UNICEF and the national survivors’ association.

The situation in the recent past has been that male survivors were being locked up, shunned and stigmatized by the community, and some chiefs also suggested that male survivors should be jailed in order to prevent sexual transmission of EVD. Upon actions from the National Ebola response committee, the attorney general wrote a letter officially condemning the persecution of male survivors. Nevertheless, male survivors are especially keen on the outcome of this study to know when it is safe to be intimate with their partners.

**1.2 Statement of the Problem and Objectives**

The purpose of this study is to assess the presence and duration of infectious EBOV in semen, vaginal fluids, and other body fluids of EVD and its relation to humoral immune response in survivors in Sierra Leone, which will help inform EBOV transmission prevention strategies during EVD outbreaks. Potential severe complications and pregnancy outcomes will also be documented.

***Hypothesis***

Infectious EBOV can persist in certain areas of the body and thus can be shed *in semen, vaginal fluids, and other body fluids in survivors,* after EBOV is no longer detectable in blood, and persistence may vary by EBOV IgM and IgG titers in serum and host factors like HIV positivity.

***Null Hypothesis***

There is no difference in the length of time that virus persists in body fluids or between individuals or in relation to antibody response or HIV status.

***1.2.1 Main objective***

To analyze EBOV persistence in body fluids (semen or vaginal secretions/breast milk/rectal swabs/saliva/sweat/urine/tears) in relation to antibody response in serum.

***Sub-objectives***

- To assess concordance between RT-PCR test results and virus isolation test results
- To characterize the genetic sequence of virus detected in body fluid specimens over time
- To assess the frequency of sequelae and severe complications at 3 and 6 months following two negative test results
- To assess if virus is detectable at long time intervals (3 and 6 months) following two negative test results
- To describe outcomes of pregnancy and delivery in pregnant participants

***1.2.2 Global framework of the Viral persistence in Ebola survivors, observational cohort study.***

**Pilot: Aims to** assess persistence of EBOV in semen among a small cohort of male survivors including assessing the feasibility of the data collection of semen.

**Main study:** Aims to assess persistence of EBOV in semen, vaginal secretions, and other body fluids (i.e. rectal, sweat, urine, saliva, tears, menstrual blood and breast milk, if any lactating women recruited) together with antibody response and HIV status among male and female EVD survivors in Sierra Leone.

***1.2.3 Specific objectives of the pilot study:***

**Pilot Study: Persistence of Ebola virus in semen among EVD survivors**

• Inform the process and implementation of the study

• Assess the feasibility of the questionnaire

• Assess feasibility of collecting semen specimen from male study participants

- Inform estimates of the range of viral persistence at different times post symptom onset in semen by specimen analysis.

***1.2.4 Specific objectives of Main study:***

**Main study: Persistence of Ebola virus in semen, vaginal secretions and other body fluids among EVD survivors**

• In 1st /baseline specimen samples, to assess period prevalence of:

a) presence of Ebola RNA detected by RT-PCR

b) of live Ebola virus detected by culture/viral isolation in: i) semen, ii) vaginal swab, iii) rectal swab, iv) sweat, v) urine, vi) saliva, and vii) tears, viii) menstrual blood and ix) breast milk, if applicable.

• To assess the maximum duration of:

a) any Ebola ribonucleic acid (RNA) detected by RT-PCR and

b) of live Ebola virus detected by culture/viral isolation in: i) semen, ii) vaginal swab, iii) rectal swab, iv) sweat, v) urine, vi) saliva, and vii) tears, viii) menstrual blood and ix) breast milk if applicable.

• To analyse the survival function, including survival probabilities (probabilities of virus persisting) at key time points, using RT-PCR and viral isolation results among individuals with specimens that are positive by RT-PCR at the baseline visit, and with subsequent specimens collected until RT–PCR and virus isolation are confirmed negative.

• To inform maximum duration of persistence in semen and to estimate a period prevalence of viral persistence in semen of survivors 12-15 months post disease onset. (‘Tail-end cohort’).

• Describe concordance between RT-PCR (including CT-values) and viral culture results for EBOV in these body fluids.

• To analyze viral persistence in relation to host factors (socio-demographics, behavioral variables, Ebola disease course and severity, other diseases including HIV, reproductive health including pregnancy, and humoral immune response.)

- To describe outcomes of pregnancy and delivery in pregnant participants (detailed in annex to the protocol)

**2. Study design**

This is a descriptive observational, bio-behavioral cohort study; no random allocation will be applied.

**Pilot study**

Entry in the study will begin with informed consent followed by a standardized questionnaire and semen collection. All survivors with a positive RT-PCR will be asked to return for a follow-up visit including repeat semen collection, after two weeks. If the second semen specimen also is positive, follow-up with repeat semen collection will thereafter take place every two weeks until the RT-PCR has been negative twice. Thereafter study participation is discontinued. The follow-up appointments will follow the same clinic flow and testing algorithms. If the initial two tests are negative, participation is discontinued. The testing interval of two weeks may be extended to 4 weeks pending on preliminary results.

Laboratory results will be shared with the participant and transmission prevention counseling and condoms provided. HIV testing and counseling will also be provided in accordance with national guidelines.

**Main study**

Entry into the study will begin with informed consent (Appendix 1a). The first study visit will include a standardized interview and collection of specimens. All interviews will take place

in a private room of the study clinic. All self-administered, blood and other body-fluid sample collections will be done in private at the study clinic, and a strict protocol for handling of bio- hazardous material will be followed. If any study participant recruited for the main study is HIV positive, s/he will instead be included in the PLHIV sub-cohort, and will be replaced in the main study.

Specimens will be tested for EBOV RNA by RT-PCR in Sierra Leone at the field laboratory. For the majority of the pilot study this took place at US-CDC field laboratory in Bo. This lab closed on 15-Oct-2015 and Chinese-CDC laboratory in Jui has now taken over the testing for the pilot study. If the US-CDC field laboratory opens in Freetown, pilot testing will return to US-CDC. For the main study, Chinese-CDC lab in Jui will conducted all RT-PCR and antibody testing. Participants with a RT-PCR positive result in any of the initial specimens will be invited to provide prospective specimens (repeat specimen collection) of each RT-PCR positive body fluid. The participant will also receive a follow-up interview at every subsequent study visit (refer to Annex 1). Once a participant has had two negative test results in a row, collection of that body fluid will stop. RT-PCR test results are estimated to be available within two weeks of specimen collection and will be shared with the study participant. Once a participant has had two consecutive negative test results for all body fluid specimens collected in this study, there will be no further follow-up. (Pending on results from the pilot study and findings of intermittent semen shedding, this may be revised to comprise more tests accordingly).

Any specimen positive for EBOV by RT-PCR will be frozen and shipped for culture/viral isolation in a BSL-4 laboratory facility at US-CDC Atlanta. The confirmatory results of viral culture will be reported back to participants when they are available.

Titers of EBOV-specific IgG and IgM antibodies in blood will be analyzed by means of serology, at the field laboratory (Chinese-CDC in Jui). Blood for antibody analysis will be collected at the same time as body fluid specimens, and will hence be discontinued when the survivor has had two EBOV RT-PCR negative body fluid specimens from each fluid sampled.

At each visit, trained counselors will provide specific risk reduction counseling on the prevention of transmission of EBOV. Participants will be offered HIV testing and will receive counseling if they elect to be tested. All detected HIV positive patients will be referred to national HIV services if not already on antiretroviral treatment. A sufficient quantity of condoms will be offered at each visit. If needed, participants will be referred to clinical survivor services. Women will be offered pregnancy testing, and if positive they will be referred to ANC, pending on gestational age and needs, care will be offered in line with national guidelines for Ebola survivors. Pregnancy is not an exclusion criteria for this study.

Pilot and main study participants will be offered a follow-up visit with a questionnaire and body fluid testing at 3 and 6 months after their second negative RT-PCR test result. During the follow-up visits, a questionnaire asking about health events that occurred since their last study visit will be given, and body fluid specimens will be collected (for the pilot, semen specimens; for the main study, venous blood, semen or vaginal/menstrual blood as appropriate, rectal swab, sweat, urine, saliva, tears, and breast milk if applicable. If all body fluid specimens test negative, no further testing will be performed until the next scheduled 3 or 6 month visit. If a body fluid specimen tests positive, follow-up testing will be performed every 2 weeks until 2 consecutive negative specimens test results are obtained.

Pregnant women will be offered a special follow-up to monitor their pregnancy and delivery outcomes and the follow-up is detailed in Annex 3 “Ebola Viral Persistence study: Nested Pregnancy cohort study - VPNP”.

**Flow charts for the main study (see below**): The first standardized interview will include demographics, sexual behaviors, and other information (refer to Annex 1). The collection of specimens will include venous blood, semen, vaginal secretions, rectal swabs, sweat, urine, saliva, tears, menstrual blood and breast milk as applicable. The participants will return after two weeks for a repeat specimen collection and a follow-up questionnaire, thereafter follow- up will take place every 2-4 weeks until all body fluids are confirmed negative twice by RT- PCR. Pending on preliminary findings in semen, the definition of a confirmed negative semen sample may be changed to comprise more tests.

**Main study structure**


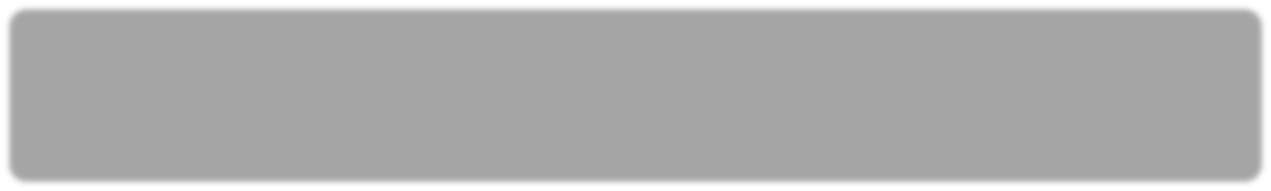

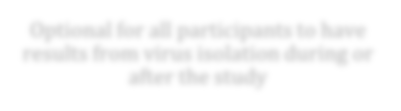

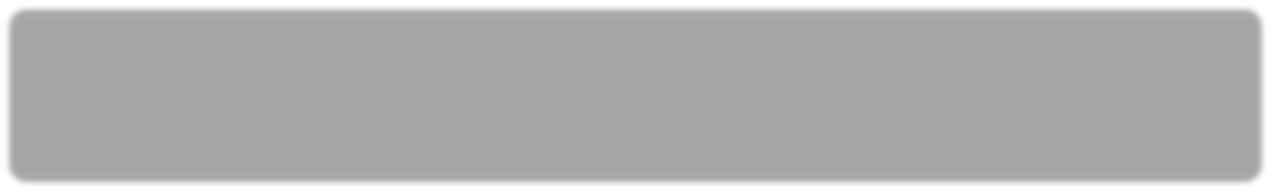

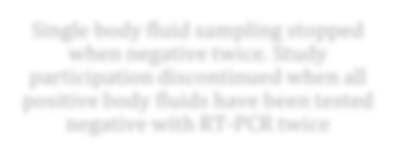

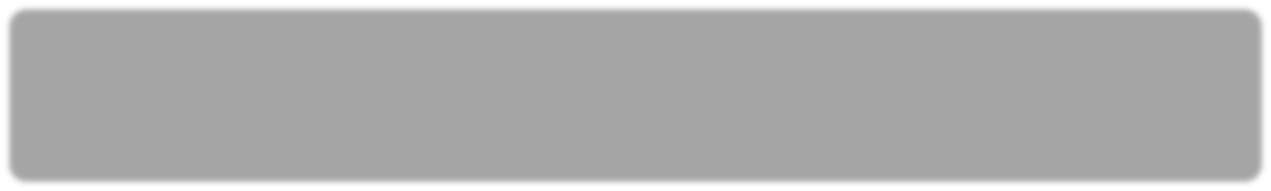

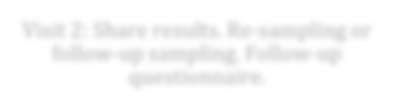

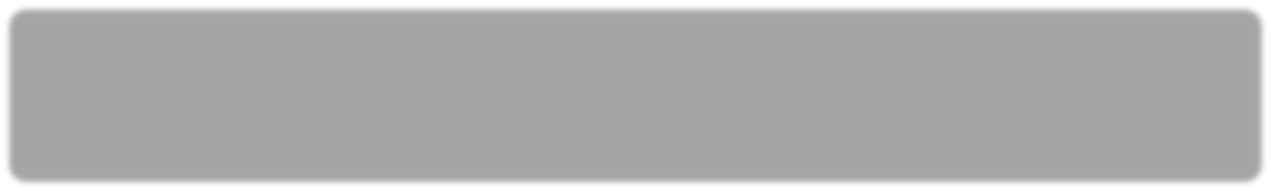

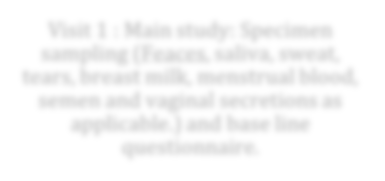

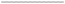

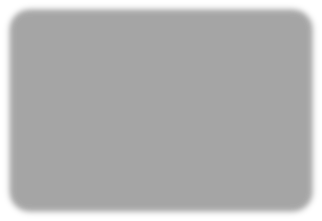

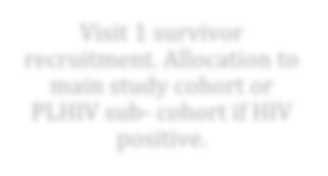

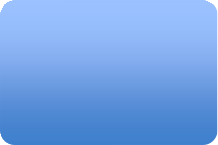

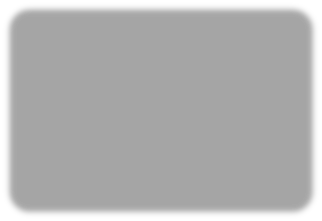

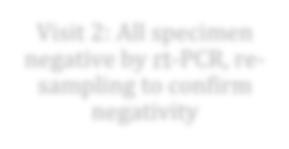

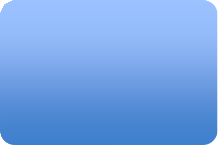

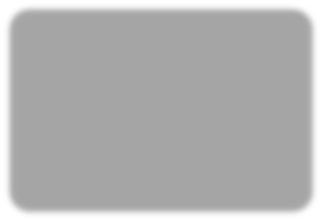

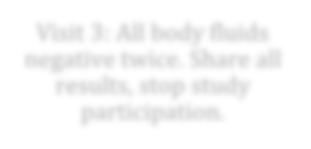

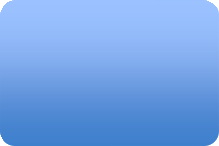

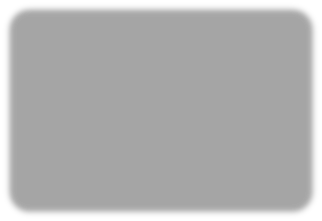

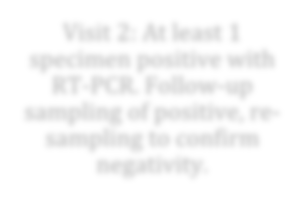

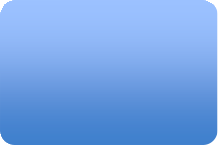

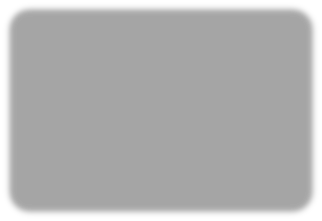

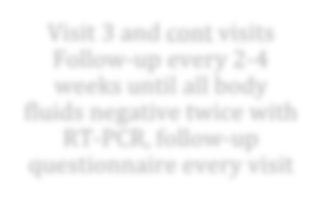

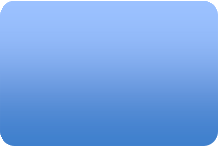

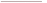


Visit 1 : Main study: Specimen sampling (Rectal swab, saliva, sweat, tears, breast milk, vaginal secretions/menstrual blood, and semen as applicable) and base line questionnaire.

Visit 1 survivor recruitment. Allocation to main study cohort, PLHIV sub- cohort if HIV positive, or pregnancy cohort if pregnant at time of enrollment.

Visit 2: Share results. Re-sampling or follow-up sampling. Follow-up questionnaire.

Single body fluid sampling stopped when negative twice. Study participation discontinued when all positive body fluids have been tested negative with RT-PCR twice

Visit 2: All specimen negative by rt-PCR, re- sampling to confirm negativity

Visit 2: At least 1 specimen positive with RT-PCR. Follow-up sampling of PCR positive specimens, re- sampling to confirm negativity of any PCR negative specimens.

Optional for all participants to have results from virus isolation during or after the study

**3- and 6-Month Follow Up Visits Flowchart**

**
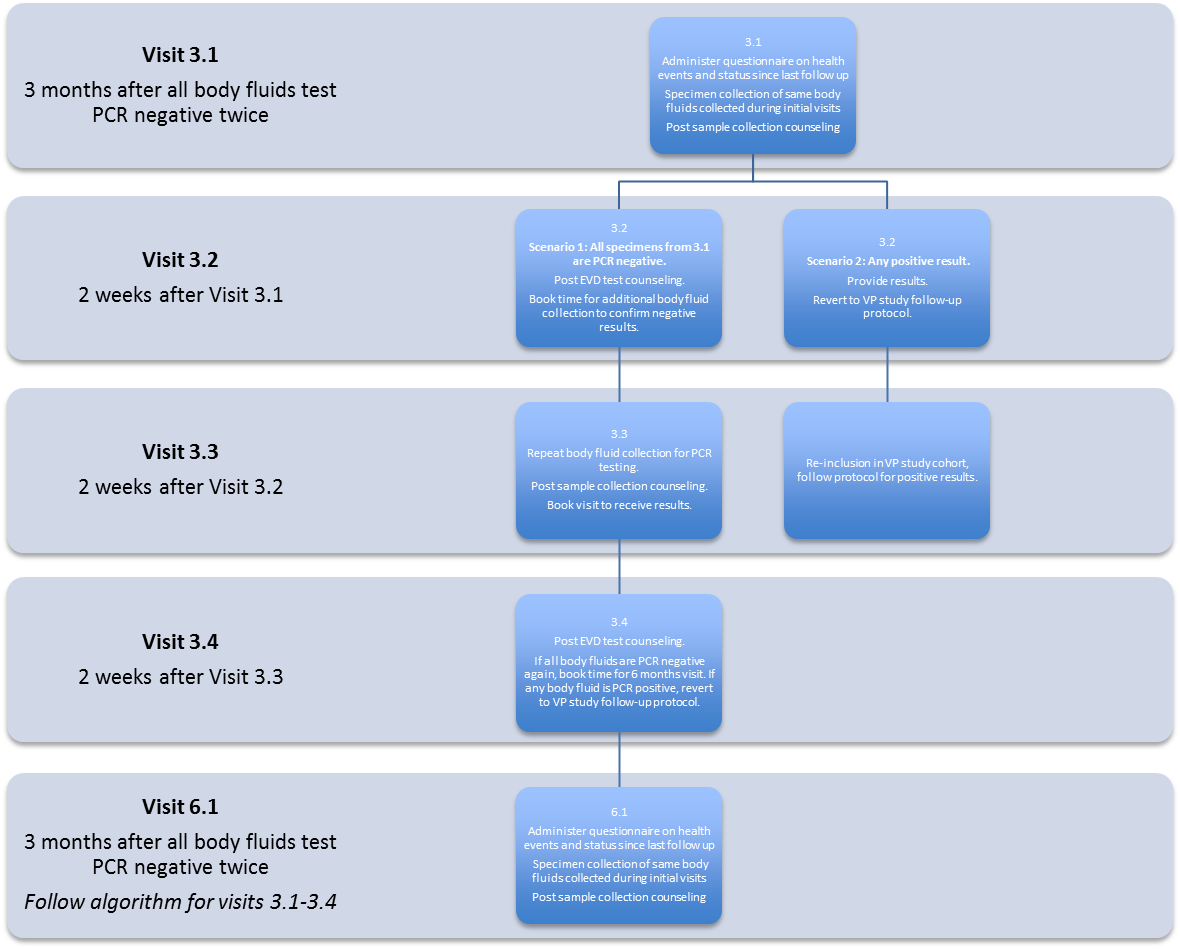
**

1. First study visit flowchart


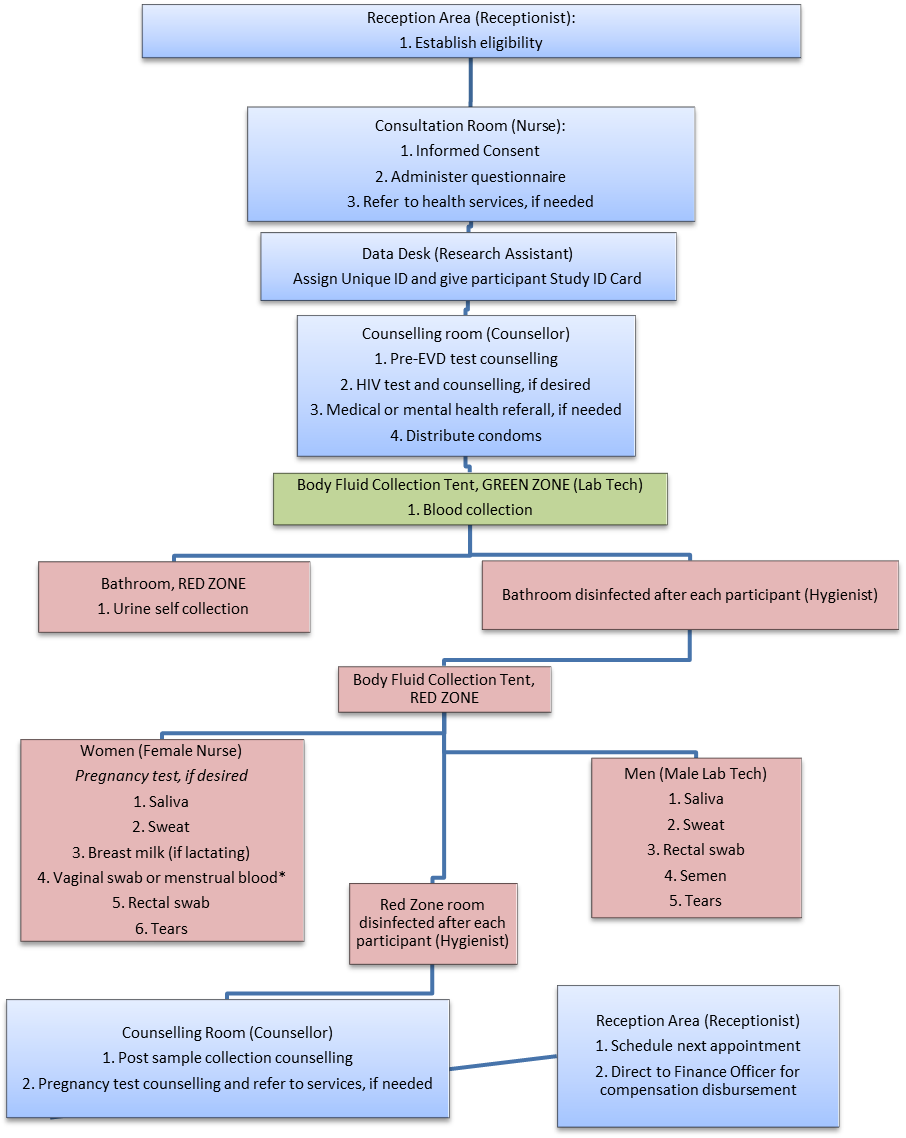


* Women should return every 2 weeks to collect all body fluids, including vaginal swab, until 2 consecutive PCR negatives. Additionally, women should come to the site during regular hours when they begin menstruating to collect menstrual blood. They do need to notify the receptionist to schedule an appointment as close as possible to the first day of their period.

2. Follow up visit flowchart


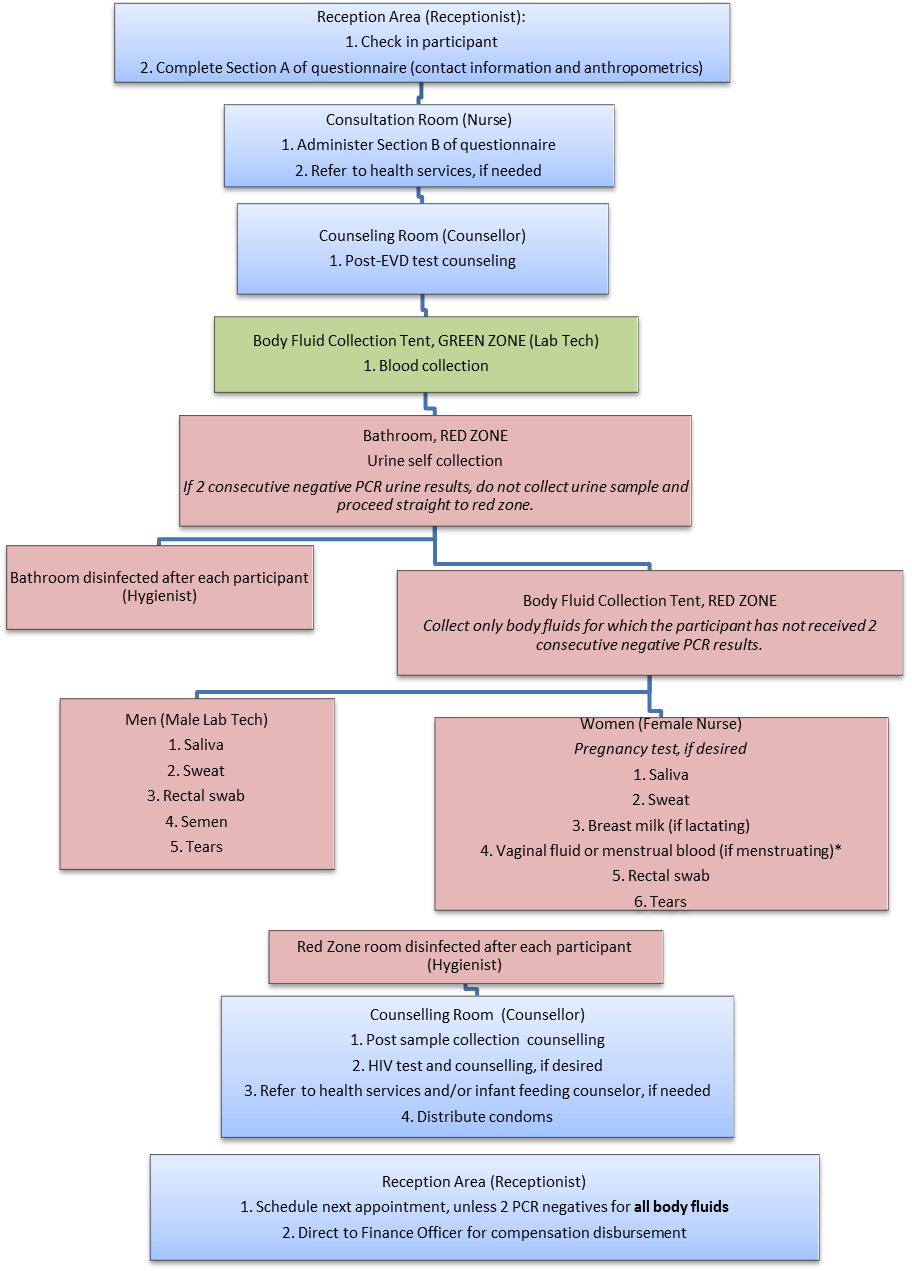


*Women should return every 2 weeks to collect all body fluids, including vaginal swab, until 2 consecutive PCR negatives. Additionally, women should come to the site during regular hours when they begin menstruating to collect menstrual blood. They do need to notify the receptionist to schedule an appointment as close as possible to the first day of their period.

**2.1 Procedures**

***2.1.1 Study site selection***

The MoHS and the Ministry of Social Welfare, Gender and Children's Affairs (MSWGCA) identified four potential study sites in three high burden districts (Western Area, Bombali, and Port Loko). To enable recruitment of recent survivors from districts with ongoing transmission, Freetown is identified as a suitable recruitment area, and the possibility of a 2nd site is under investigation. Preliminary information suggests that survivors treated in Kenema during the early phase of the epidemic may also be residents of Port Loko, since no ETU was established there initially.

The pilot study has been conducted at M34 in Freetown. The main study will continue at M34 in Freetown.

In addition the possibility to start up a 2nd study site is being investigated. Guiding principles for selection of a 2nd site are feasibility in terms of construction and staffing and availability to invite more recent survivors, as well as tail-end long term male survivors. Lungi Government Hospital, as well as other options such as expansion within the MoD network of health posts, are currently explored.

The study sites will be the setting for all initial and follow-up visits of survivor participants and the location for interviews, specimen collection, and risk reduction counseling regarding test results. Study participants will be referred to HIV and survivor services within the hospitals where the study sites are located.

**Specimen storage, transport and testing**

• All specimens will be kept cold and transported to the field laboratory for RT-PCR testing within 48 hours.

• Transport will be organized three times a week from the M34 hospital during the pilot study, and then daily from the study sites to the field laboratory.

• Transport routines will follow guidelines on safe handling and transportation of hazardous bio-materials, which are further detailed in page 70+ and in: [Guidance on regulations for the Transport of Infectious Substances](http://www.who.int/ihr/publications/who_hse_ihr_2012.12/en/)

• At the field laboratory, specimens will be split into three aliquots: one for RT-PCR, and two for frozen storage.

• Aliquots of specimens testing positive by RT-PCR will be appropriately packaged, labelled as Category I biohazards, and shipped internationally under appropriate infection control precautions to CDC in Atlanta, GA, USA. The remaining specimen aliquot will remain in frozen storage in Sierra Leone.

• Virus isolation will be performed outside Sierra Leone, at a BSL-4 laboratory facility (US-CDC Atlanta).

• Blood for further analyses by immunofluorescence assays will be stored at room temperature and transported for analyses within 48 hours.

***2.1.2 Study participants***

***A. Participant recruitment***

Survivor associations in each of the districts will be involved with the study implementation. A small group of survivors will be formed to serve as key informants for sensitization of the community and recruitment for the study.

In collaboration with the survivors association and the MSWGCA, a list of established EVD survivors will be selected in accordance with sample size criteria. These groups of established survivors will be invited to a meeting in their district where they will be informed about the study process and benefits by MSWGCA staff and/or survivor key informants. The interested participants will be asked to provide contact information and an appointment will be scheduled for their first study visit. In addition print and radio advertisements may be used to recruit for the study.

In addition, people living with HIV (PLHIV) who also are Ebola survivors will be invited to participate in a sub-cohort. The “network of HIV positives in Sierra Leone” (NETHIPS) and local networks who work with PLHV will be used to identify and reach out to participants. HIV counsellors will also encourage their clients who are EVD survivors to participate in the study.

**Pilot**

The recruitment of survivors for the pilot will be done on a voluntary basis with the help of the association of survivors in Freetown and before the sample for the main study is selected. A collaboration with the national survivors’ association has already been started and the organisation has been part of the formative work. The association foresees no challenges in recruiting participants for the pilot study.

**Main study**

Recruitment from

the ETUs

All adult patients discharged from an ETU during the recruitment period will be asked to participate in the study

Recruitment from survivor registry

Survivors will be contacted according to their duration of convalescence and their place of residence.

Recruitment from other sources

If the number of patients is not enough, an open cohort process where participants will be recruited by using local ETU records, survivor networks and services; community outreach, including radio solicitations, may also be considered.

PLHIV networks PLHIV EVD survivors will be contacted by PLHIV support groups and networks in addition to HIV counselors who were closely with them

***B. Participant enrollment***

General inclusion criteria are adults (>18) who have survived EVD, with time lags since disease further specified below. Survivors with experiences of experimental treatment during their EVD as well as any pregnant women will be included in the study.

Eligible study participants must demonstrate an EVD discharge certificate and a national photo identification card in order to be recruited.

In order to ensure survivor status and eligibility for the study, we may cross-check laboratory information as available.

Participants will receive a study ID card that will be used for verification of identity at subsequent study visits.

Exclusion criteria are age < 18, no verified EVD discharge certificate, inability, for any reason, to follow study information and consent procedures.

**Pilot**

Adult male (18 years or above) survivors will be enrolled if eligible, with assistance of the survivors association in Freetown and the collaborating hospital and clinic.

**Main study**

All adult men or women (18 years or above), discharged from an ETU during relevant time periods (please see sample distribution), holding a certificate of recovery, are eligible.

***Sample size calculations***

**Pilot study sample size:**

A maximum of 100 men (not based on sample size calculations in relation to power, but on feasibility for assessing study process and generating enough knowledge for main study) will be included with a target of 40% between 2 and 3 months convalescence period and 60% with over 3 months convalescence period. These men will be followed until they have provided two consecutive negative semen specimens within a 2 – 4 week interval, and may be merged with the main study cohort as needed.

**Main study sample size:**

The following is the rationale for estimating the sample size needed for body fluids other than semen, when hypothetically the prevalence of persistence will be low and approaching zero, and when there is a wish to ‘rule out’ the possibility of viral persistence at a given time point by way of independent specimen provided by participants at baseline. The below sample size calculations for a zero prevalence estimate hence refer to estimating a population proportion at a certain point (or period) in time (i.e. at a specified time since EVD onset).

For the primary outcome of live virus isolated in tissue culture, even one positive (live EBOV virus) outcome is of interest; as such a finding would be expected to have important implications for guidance to future caregivers, patients, and their families. Similarly, we would not want to conclude that there is no probability of persistent live EBOV virus in the fluids we test if, in reality (within the larger population), there is. Therefore, the primary power calculation of interest is the degree of confidence we can have in a null (no positives) result from our tissue culture testing. In other words, we need to know the upper confidence limit for a calculated proportion of 0% in our study.

There are several ways to calculate an upper bound for a 95% confidence interval for a 0% finding. The rule of thumb for the upper bound is 3/N, where N is the number of specimens tested.6 This method is accurate when the sample is sufficiently large. Calculating the upper bound precisely requires use of the binomial distribution. The table below lists the upper bounds for a 1-sided 95% confidence interval estimated using Stata SE V12.1

Numerator 0 0 0 0

Denominator 50 100 200 300

Upper bound 7.1% 3.6% 1.8% 1.2%

Lower bound 0% 0% 0% 0%

In line with the above, based on the assumption of a 1st sample period prevalence (0-3 months) of viral persistence in body fluids other than semen, approaching zero we aim for a sample of 100 total participants (50 men and 50 women), adding an additional 20% of participants to account for potential drop-out. We would then arrive at a 95% confidence interval in gender specific sub-analysis of 0-7.1% and if all specimens are negative for both sexes a collapsed group analysis with a 95% CI of 0-3.6%.

**Main study - Sample size rationale:**

| **Cohort** | **Male** | **Female** | **TOTAL** |
| --- | --- | --- | --- |
| Recent survivors  (0-3 months post-symptom onset) | 120 | 120  • including menstrual blood samples, as available  • including breast milk samples if any lactating women are enrolled | 240 |
| PLHIV |  |  | 30 |
| **TOTAL** |  |  | **270** |

**1. 60 men and 60 women (50+20% drop-out) should hence be included, in each of the study sites (Freetown and Porto Loko.)**

In order to maximize the possibility of detecting virus RNA in body fluid specimens, the lag time since symptom onset should be pushed towards recent survival (3 months post discharge or less). If male or female survivors with <= 3 months since discharge cannot be identified, the lag time may need to be postponed until 6 months post discharge. Male survivors from the pilot study may be invited for this purpose, for additional body fluid testing, but would need to be drawn from a random list of pilot participants, giving semen negative men an equal probability as semen positive to participate.

**2. Menstrual blood:**

For the purpose of collecting menstrual blood, a sample of 50 women would be needed, if the prevalence approaches zero, in order to arrive at a 95% CI 0f 0-

7.1%. All of the 60 women of the main study will be informed and invited to provide specimen from the first or initial days of the menstruation period. It is expected that not all of these women would be regularly menstruating and able or willing to participate. Through the same channels as above, women survivors, of preference up to 3 months post-discharge, else up to 6 months, will be invited to cover the sample size needed.

**3. HIV positive Ebola survivors’ sub-cohort:**

The expected number of PLHIV who have also survived Ebola is supposedly low. NETHIPS has done an estimate based on their network, by district, suggesting some 30 PLHIV Ebola survivors in total in the country. NETHIPS and other relevant networks and resources will be used to invite participants; these participants are most likely already on ART. HIV Counselors will also be engaged to encourage their clients who are EVD survivors to participate in the study; these participants may not yet be on ART. All identified PLHIV who have survived Ebola would be included in this sub-cohort, up to a maximum of 50 men and 50 women, irrespective of time since discharge for Ebola, and also from all geographical regions, as feasible. These PLHIV will constitute a separate sub-cohort. If any study participant recruited for the main cohort is presenting with a known HIV infection or is diagnosed with an HIV infection within the study, s/he will be included in the PLHIV sub- cohort and will be replaced in the main study cohort.

**2.2 Admission procedure**

**Main study**

1. For all patients recruited, the study will be explained and informed consent obtained and signed (see Section 2.1.2 on patient enrollment and Appendix 1; see Ethics section below). Contact information will be collected.

2. At the first visit, the participant will be assigned an individual personal ID number for the study. A study ID card should be given to the patient with his/her ID number. In a secure electronic record, linking sex, age, name, chiefdom, ETU case report form (CRF) number and survivor certificate numbers (if available) to the ID number should be kept safely and only used for returning study participants who lost their ID card. A designated study team member will record information on exposure to experimental treatment that was captured in the participants corresponding ETU CRF where available).

3. A base-line interview with the survivor by way of a standardized questionnaire (refer to Annex 1) including questions on socio-demographics, symptom debut, co- morbidity, house-hold members, menstruation, breast feeding and current sexual partners will be done at the baseline and at any follow-up visits. The interview will be conducted by a trained interviewer from the MSWGCA. The questionnaire will be available in English, Krio and any local languages in the catchment area.

4. After the administration of the questionnaire, there will also be the1st sampling of body fluid specimens. Specimens of semen and urine will be self-collected by the participants. Rectal swab, vaginal secretions, and breast milk specimens will also be self-collected or collected with assistance from the lab technician or nurse. Sweat, saliva, and tears will be collected by the lab technician or nurse. A venous blood sample will be drawn to enable analysis of EBOV specific IgM and IgG antibody titers in serum. Procedures for specimen collection will be explained by study staff (see operations manual) and specimen collection will be completed in a secure, private space in the study clinic using appropriate infection control precautions.

5. The participant will be offered EVD test results from prior study visits and standardized counselling on Ebola transmission risks by trained personnel of the same sex as the participant. Participants will be provided with a 5-week supply of condoms (approximately 35 condoms). Women with positive breast milk will be given infant formula at every visit. Participants will be provided with a financial incentive for participation in each study visit (Section 12.4). Additionally, the follow-up visit for provision of test results will also be scheduled and all contact information will be reviewed again.

6. Participants will be given the option to receive an HIV test and pre and post-test counseling. All HIV positive patients will be referred for HIV follow-up care as per national guidelines. Participants who are known to be HIV-infected will be offered counselling on living with HIV and linkage to care via the Sierra Leone national AIDS control program if they are not currently engaged in HIV care.

7. Participants who are HIV positive will be included in the PLHIV sub-cohort. A confirmatory HIV test will be performed at the time of the first visit.

8. Women will be offered pregnancy testing, and if positive they will be referred to antenatal care (ANC), pending on gestational age and needs, care will be offered in line with national guidelines for Ebola survivors. If a woman states that she knows she is pregnant, a confirmatory pregnancy test will be performed.

9. Participants will be offered their RT-PCR test results at the subsequent study visit, 2 weeks after the current one.

10. Risk reductionounseling about avoiding potential transmission of Ebola will be provided to all participants as part of pre-test and post-test sessions, and their test results will be provided along with individualized counseling messages.

**Specimen Collection Main study**

1. Specimen collection will take place in designated areas of the study clinic, in a special room designated for this purpose. The study technician will label and mark all specimen containers before collecting, with the participant’s personal ID number, study name, visit number, date, specimen description and name of clinic. All specimen collection will follow guidelines; see further details in operations manual.

2. Blood, sweat, tears and saliva will be collected by a trained healthcare worker. Breast milk, vaginal swab (for vaginal secretions and menstrual blood) and rectal swab can either be self-collected, or collected by a gender appropriate, trained healthcare worker. Semen and urine will be self-collected by the participant using low- literacy illustrated instruction cards and detailed verbal instructions from the trained healthcare worker. Specimens placed on a designated counter in the private room. A trained healthcare worker (well informed of the SOP and trained in infection control procedures and wearing adapted personal protection equipment (face mask, apron, gloves, and boots) against potential contaminated fluids) will then enter the room and seal the container with the specimen respecting all infection control procedures.

3. Serum will be collected by a trained healthcare worker, the procedure aligned with standard practice for infection control.

4. For male participants, urine should be collected prior to semen specimen collection to prevent the risk of contamination.

5. Collection of the different specimens will follow standard operating procedures (Outlined in operations manual).

Specimen projections by visit, MEN 1-3 months post EVD symptom onset:

|  | **Blood** | **Semen** | **Saliva** | **Tears** | **Urine** | **Sweat** | **Rectal** | **TOTAL** |
| --- | --- | --- | --- | --- | --- | --- | --- | --- |
| **Visit 1** | 120 | 120 | 120 | 120 | 120 | 120 | 120 | 840 |
| **Visit 2** | 120 | 120 | 120 | 120 | 120 | 120 | 120 | 840 |
| **Visit 3** | 120 | 120 | 120 | 120 | 120 | 120 | 120 | 840 |
| **TOTAL** | 360 | 360 | 360 | 360 | 360 | 360 | 360 | 2520 |

Specimen projections by visit, WOMEN 1-3 months post EVD symptom onset:

|  | **Blood** | **Vaginal OR Menstrual Blood** | **Saliva** | **Tears** | **Urine** | **Sweat** | **Rectal** | **Breast Milk** | **TOTAL** |
| --- | --- | --- | --- | --- | --- | --- | --- | --- | --- |
| **Visit 1** | 120 | 120 | 120 | 120 | 120 | 120 | 120 | ? | 840 |
| **Visit 2** | 120 | 120 | 120 | 120 | 120 | 120 | 120 | ? | 840 |
| **Visit 3** | 120 | 120 | 120 | 120 | 120 | 120 | 120 |  | 840 |
| **TOTAL** | 360 | 360 | 360 | 360 | 360 | 360 | 360 |  | 2520 |

Projected number of samples from men and women for:

• First 6 weeks of study: 5040 specimens

• 4 month period: 3840 specimens

• Assuming approximately 6 participants per day with an average of 7 specimens per participant, 42 specimens per day / 240 specimens per week

• Handling and transport will follow routines for safety and transport as outlined in operations manual.

• The examination room will be cleaned after use by sanitation workers (hygienist) wearing standard protection against potential contaminated fluids.

***2.2.1 Follow-up procedures***

**Main study**

The EVD survivors with at least one of the body fluid positive at RT-PCR will be asked to return for follow-up study visits, first time after two weeks then every two to four weeks until there have been two consecutive negative RT-PCR test results for each body fluid. Participants will be asked to continue follow-up until all body fluid specimens are negative for two consecutive visits.

Lactating women will receive their results within 3 days after the collection of the specimen. Women with positive results in breast-milk will be offered testing every 3 days till 2 consecutive tests are negative; women with a first negative test will come back after 3 days to do a confirmatory test.

Menstruating women will be asked to provide a specimen as close to the first day of their period as possible, and a second specimen before the end of the period. Menstrual blood sampling may be done at the same time as other body fluid testing, or may occur after testing of other body fluids has been completed.

Women will be offered pregnancy testing at every visit, and if positive they will be referred to ANC, pending on gestational age and needs, care will be offered in line with national guidelines for Ebola survivors. Pregnant women will be offered participation in the study.

Pregnant women will have an additional follow up questionnaire to record the outcome of delivery, which will occur if a pregnant woman delivers before she has completed the study follow up, or if the delivery takes place after body fluid testing has finished. A special questionnaire to record the pregnancy and delivery outcomes will be completed by the healthcare personnel providing ANC.

Breast-feeding women who test positive for EVD in breastmilk will be offered free formula feeding in line with current WHO guidelines.

A study card ID check will be done for each participant coming for a follow-up visit. If the participant has lost his/her study ID card, the locked in ID code may be accessed, if participant’s identity can be verified.

At each visit, participants will be informed of their RT-PCR Ebola test results (if available) including negative results. HIV testing will be offered at each visit. A follow-up questionnaire will be administered, including sexual behavior over the last two weeks since the previous visit, see Annex 1.

Participants will be asked to continue providing specimens of each body fluid and venous blood at every study visit until they have had two consecutive negative test results for that body fluid. Participants whose body fluids all have been RT-PCR negative at two consecutive study visits will exit from the study cohort.

For participants that have had two consecutive negative test results, a follow-up visit to administer a questionnaire and body fluid testing will be scheduled at 3 and 6 months after the last 2^nd^ negative body fluid specimen has been collected. The same body fluids would be collected as in the initial visits, and the questionnaire will focus on health events and status since the last follow-up visit.

Interviews will take place in a designated private room at the study clinic.

A time to come back to the clinic for follow-up will be booked and agreed upon at each preceding visit. Contact information will be reviewed at each visit and reminder calls will be made if applicable. Study participants will be supported to fulfill their participation in the study by counseling offered at each visit together with assistance with referrals to clinical care as needed. Carefully timed reminder calls will be made to participants who have missed their appointments, and flexibility will be provided in terms of appointment bookings and any needs for additional counseling or information.

Renumeration for participation will be paid at each visit.

The participant will be offered test results from the preceding visits and offered counselling on transmission risks targeted to lab findings according to a standardized counseling script (Annex 2).

***2.2.2 Criteria for discontinuation of a participant***

1. The prospective data collection from a participant including specimen collection in the Main study will be discontinued when specimens from all relevant body fluids are negative (two negative RT-PCR test results at consecutive study visits constitute a negative, or else amended as per preliminary results.)
2. If the specimens test negative at the 3 and 6 month follow-up visits, no further visits will occur.
3. If male participants are unable to provide a semen specimen at 2 consecutive visits, they will be referred to specialized urological care via a survivor clinic. Additional eligible survivors may be recruited to replace these participants.

***2.2.3 Laboratory and other investigations***

All laboratory results will be filled into a form marked with the participant’s study ID and kept in confidential storage for entering of follow-up results.

**At the study site:**

- All biological specimens will be kept cold and will be transported from the study sites to the US-CDC Bo laboratory (three times weekly during the pilot study) and to Chinese-CDC Jui laboratory at least three times weekly for the main study.

- All sampling and handling of specimens collected for transportation should follow procedures for labeling, packaging, transporting, and storing bio-hazardous material detailed in the operations manual.

**At the Field laboratory (US-CDC BO for Pilot, Chinese-CDC Jui for Main study):**

- For the pilot study, RT-PCR will be performed on all specimens for two viral targets, VP40 and NP, as well as an internal human control, B2M, according to guidelines detailed in appendices 5 and 6. For the main study, conducted at Chinese-CDC, the two RT-PCR gene targets are NP and GP, but the internal control remains the same.

- If the internal human control is negative for a given specimen, this would be viewed as an inadequate sample and a new specimen would be requested.

- Specimens will be stored at -80°C and those that are RT-PCR positive will be shipped to a BSL-4 laboratory for virus isolation testing (US-CDC Atlanta except the semen for the main study), following appropriate protocols for transporting Category I biohazards

- Blood specimens collected during the main study will be tested using an Enzyme Linked Immunosorbent Assay to assess IgG and IgM antibody presence and titers.

**US-CDC Atlanta BSL-4 laboratory:**

- In order to determine if a given body fluid specimen has live virus and is potentially infectious, virus culture will be performed. This can only be safely performed in Biosafety Level (BSL)-4 laboratory conditions, which are not available in Sierra Leone. Therefore specimens which have detectable EBOV RNA by RT-PCR from the pilot study will be transported frozen with adequate safe packaging and cold chain to Atlanta, GA, USA for virus isolation.

- In addition to positive specimen from the pilot study being tested at US-CDC Atlanta, additional virus isolation of body fluids other than semen from the main study will be done in Atlanta.

- For the pilot study, testing for live virus will be performed at the US-CDC BSL-4 laboratory in Atlanta, GA, USA, ideally within 6 months of specimen receipt. Standard EBOV virus isolation protocols for diagnostic specimens will be followed.

- If specimen type and volume permit, the viral titer (TCID50/mL) for the specimen will be determined. This will inform the amount of infectious virus in a specimen, unlike virus isolation which will tell you a binary, (i.e., yes or no) to infectious EBOV virus in the specimen.

- Residual body fluid specimens will be destroyed. Viral isolates, if any, may be preserved at US-CDC for further study in collaboration with MoHS and WHO (in accordance with the research collaboration agreement). Body fluid isolates will be kept in liquid Nitrogen frozen storage (-165oC) in a secure location at US-CDC’s BSL-4 laboratory. Virus isolates can be shared to requesting parties assuming that the requesters can comply with the necessary biosafety requirements (BioSafety Level-4, Select Agent compliance). Further study of virus specimens may include genetic analysis and comparison with other virus strains.

**3. Study instruments**

(See Equipment and Supplies in operations manual)

**4. Project management**

This project is led by the Sierra Leone MoHS and MSWGCA, under Principal Investigator (PI) Dr. Gibrilla Fadlu Deen, in collaboration with co-investigators from WHO, US-CDC and Chinese CDC respectively.

| **Ministry of Health and Sanitation (MoHS)**  **Ministry of Defense (MoD)**  **Ministry of Social Welfare, Gender, Children’s Affairs (MSWGCA)** | **PI:** Dr Gibrilla Fadlu Deen  **Co-PIs:** Dr Amara Jambai, Dr Alie Wurie  **PI (nested pregnancy cohort):** Dr T George    **Site coordinators:** Col (Dr) Foday Sahr, Lt (Dr) Foday Sesay, Cpt (Dr) Massaquoi  **Additional Investigators:** Abdul Kamara, Tina Davies |
| --- | --- |
| **World Health Organization** | **Coordination (Geneva):**  Sexually transmitted infection, Epidemiology subject matter expert:  Dr Nathalie Broutet  Dr Anna Thorson  **Pregnancy cohort:** Dr Olufemi Oladopo  **Coordination (Sierra Leone):**  Dr Suzanna McDonald  Dr Philippe Gaillard (Field Study Coordinator)  **Laboratory/Virology**: Dr Pierre Formenty  **Research collaborators/Implementation:** Ms Kara Durski  Ms Jaclyn Marrinan  Dr Teodora Wi  Dr Faiqa Ebrahim  **WHO Sierra Leone**:  Dr Mauricio Calderon  Dr Margaret Lamunu  **Reproductive health experts**:  Dr James Kiarie (Coordinator RHR),  Prof Marleen Temmerman (Director RHR)  Dr Lisa Thomas MD |
| **US Centers for Disease Control and Prevention** | **Atlanta, GA:**  Ebola Epidemiology subject matter expert: Dr Barbara Knust  Subject matter experts, sexually transmitted disease epidemiology research:  Dr Kyle Bernstein  Dr Elissa Meites  Subject matter expert, sexually transmitted disease behavioral science:  Dr Neetu Abad  Ebola laboratory subject matter experts:  Ute Stroeher  Bobbie Rae Erickson  Additional staff: Dr. Eloisa Llata, Deborah Dee, Shacara Johnson, Neil Rainford, Michael Fox, Rachel Kaufmann  **Sierra Leone:**  Dr Oliver Morgan  Dr Sarah Bennett  Dr Dianna Ng  Dr Christine Ross  Ms Elizabeth Ervin  Ms Tara Sealy |
| **Chinese Centre for Disease Control and Prevention** | Dr Wenbo Xu  *Team Lead of China P3 Lab, Chief of National Laboratory of Measles and Acting Chief of National Laboratory of Poliomyelitis, Deputy Director of National Institute for Viral Disease Control and*  *Prevention*  Dr Hongtu Liu  *Professor and Chief of Department of Tumor-Associated Virus National Institute for Viral Disease Control and Prevention Chinese*  Dr Wenjiao Yin |
| **UNAIDS** | Ms Patricia Ongpin |

**4.1 Staffing and work plan**

Staff needed per study site:

- Study supervision: medical superintendent of each hospital where study site located

- Study coordinator: study physician from the hospital

- Three nurses for enrollment, interview, sample collection instruction, HTC

- Lab technician for collection of samples, sample packaging and transport

- Two counselors for pre and post EVD and HIV test counselling: one is a psychiatric nurse and the other is a nurse with a background on training for HIV counselling

- Driver

- Two community liaison officers (Ebola survivors) for community sensitization, recruitment, appointment scheduling

- Two Hygeinists to clean the specimen collection areas and study site
- Research Assistant to enter questionnaire data, generate laboratory line list, and assign Study ID numbers
- HR/ Administration Coordinator
- Data Management Coordinator/Data analyst

***Capacity Building:***

With the exception of the study coordinator and operations manager, both the pilot and main study will be staffed by local staff identified by the implementing partner, with special effort to employ and train Ebola survivors in research skills. Staff will receive training in counselling, community outreach, HTC, data management, research methods, and IPC.

The laboratory partner, Chinese CDC, runs a 2-week capacity building training course for local laboratory staff covering laboratory techniques. As part of this research partnership, Chinese CDC will train and employ local laboratory staff.

**4.2 Administration and monitoring**

The on-site project manager will administer the project under the overall guidance of the PI and partners. The study team may use workflow assignment (or Gantt) charts to help monitor progress. Regular meetings will be held, and progress reports on major results of interviews or analysis will be written at least monthly. Delegated team members will monitor the quality of the data, teamwork output as well as compliance with the project work schedule.

An external monitor, Dr Ivan Velez, has been invited by the WHO to perform a monitoring visit at the pilot study site in August 2015.

**5. Data quality assurance**

Only trained interviewers and/or lab assistants will be used for the interviews and specimen collection. The topics included in the questionnaires include asking for information that may be perceived as sensitive and efforts will be made to ensure a private setting and calm environment to assure internal validity of the data collected.

Handling and treatment of biological sampling and specimen will follow standard operating procedures. Specimens sent for viral isolation will be handled at appropriate laboratories following relevant protocols for potential biological hazards.

Study staff will review data collected and entered for quality and consistency. Data quality reports will be developed and generated weekly with feedback to study staff at the lab and participating ETU sites.

**6. Data management**

• All MoHS-owned data related to a study participant will be assigned the participant’s unique study ID. No patient-identifying information (i.e., names) will leave Sierra Leone.

• Interview data will be organized in databases stored on secure local servers within the Ministry of Health in Sierra Leone and WHO and will be backed-up regularly. Collected data will be directly recorded on computers or tablets to minimize data recording and entry errors and minimize delays in data availability. If paper forms must be used, interview responses will be entered into the database either daily or as a group at the close of data collection; and 10% of entered forms will be re- checked to identify any problems with data entry accuracy that must be addressed.

• Electronic equipment and files will be kept password-protected.

• Paper forms and electronic devices will be kept locked when not in use.

• All individual data identifying direct patient identifiers will be removed from the dataset before analysis and replaced with a unique participant code that can be linked back to individuals via a master key at a centralized secure server and database.

• Individual records and the key linking the participant code number will be kept secure, accessible only to the local study team under the supervision of the study PI and MoHS in collaboration with WHO, US-CDC and Chinese-CDC.

• Paper interview forms, if used, will be destroyed within one year after all data are entered and verified.

• Laboratory RT-PCR results will be batch processed and complete RT-PCR results for all specimen types will be reported back within 1 week of specimen receipt to each site coordinator. A positive RT-PCR result on any specimen should be entered in the secured data base and reported within 8 hours to the study PI and WHO, US-CDC, Chinese-CDC local coordinators as well as the site coordinator where the specimen was collected.

- Antibody testing will be batch processed and results will not be reported to participants.

• Laboratory staff will identify specimens only by the labelled study ID, and will not have access to any personally identifying information.

• Counsellors will provide laboratory results only in person and only to participants who (1) return for a follow-up study visit; and (2) present their study ID card or confirm their identity and study ID number; and (3) agree to receive their own results. Individual results will not be shared with anyone other than the study participant. Results will be presented according to the counselling script.

• Virus isolation results from US-CDC Atlanta will be reported promptly to the study PI and entered in the secured data base as they arrive. These confirmatory results will not be available within a clinically relevant time frame (6 months). Positive results will be reported to participants.

**7. Data analysis plan**

• Descriptive analysis of the study participants will be performed by the study PI and staff with the support of WHO and US-CDC. WHO and US-CDC technical assistance may be provided as needed for these and any additional analyses of the study data. Any further statistical analysis will be conducted using adequate statistical program packages with the assistance of WHO and US-CDC.

• Viral Persistence time will be measured in days or months from time after symptom onset and will also be presented as time after ETU discharge.

• The primary measures of interest are:

o the period prevalence of viral persistence in 1st/baseline samples (0-3 months, >3-6 months etc)

o survival probabilities over time (based on base-line positive samples)

o maximum duration of tissue culture-positive results for EBOV by number of days since symptom onset for each of the various body fluid specimen types.

• EBOV presence, measured both as detected RNA and isolated virus, in any of the relevant body fluids, will be analyzed in relation to time of persistence.

• Analysis of RNA derived from selected specimens collected during the study will include genetic analysis and comparison with Ebola virus sequences derived from the participant. This will include sequences characterized during the acute phase of EVD (if available) and comparison with other Ebola virus sequences that have already been characterized. This will help to better describe the variation in virus sequence over time within individuals.

• Viral persistence in a body fluid at a certain period in time, will be analyzed in relation to host factors such as age, sex, co-morbidity including HIV and its clinical characteristics, severity of the earlier Ebola infection and Ebola sequelae, as well as presence and titers of IgM and IgG antibodies in serum. Information on exposure to experimental treatment or vaccination will also be taken into account in the analysis.

• Participant information on sexual history will be analyzed for frequency and type of

(protected/unprotected/vaginal/oral/anal) sex.

• The calculation of correlation between positive RT-PCR and culture tests for each body fluid specimen types will be performed, conditioned upon time since the first convalescent negative RT-PCR blood test.

• ETU CRFs will be accessed retrospectively to investigate clinical severity of disease and so on. Currently we do not know what the quality of the data in the ETU CRFs will be, as they have not been accessed. Where ETU records do exist for study participants, there may be inconsistency in the data collected between the ETU CRFs and the study questionnaires. The ETU CRFs will primarily be used to add additional information that the participant may not have been able to recall; and verify data that has been collected in the corresponding section of the questionnaire. In cases where there are discrepancies with what is recorded in the questionnaire and the ETU CRFs, additional weight will be given to data captured in the questionnaire. The data capture for the study questionnaires is conducted by designated, trained team members and is therefore standardised. Data collected in ETU CRFs is likely to have varied between ETUs and is likely to have been collected in a less uniform way (unknown amount of training of staff, likely to have been collected by a large pool of staff and so on.

- 3 and 6 month visit body fluid RT-PCR test results will be analyzed to describe if body fluid specimens can test positive after two consecutive negative test results. A descriptive analysis of the questionnaire will be performed to assess any clinical complaints or health events, and to compare them to previous test results for any correlation of complications related to previously detected virus persistence.
- Outcomes of pregnancy will be described and related to the participant’s previous test results.

**8. Work plan and study timeline**

| **ACTIVITIES**  *Lead Agency* | **STUDY TIMELINE** | | | | | | | | | | | | | | | |  | | | |
| --- | --- | --- | --- | --- | --- | --- | --- | --- | --- | --- | --- | --- | --- | --- | --- | --- | --- | --- | --- | --- |
|  | **2015** | | | **2016** | | | | | | | | | | | | |  | | | |
|  | *Summer* | *Fall* | *December* | *January* | *February* | *March* | *April* | *May* | *June* | *July* | *August* | *September* | *October* | *November* | | *December* |  | | | |
| Local ethics committee review  *MoHS* |  |  |  |  |  |  |  |  |  |  |  |  |  |  | |  |  | | | |
| Investigators coordination meeting  *MoHS* | **X** |  |  |  |  |  |  |  |  |  |  |  |  |  | |  |  | | | |
| Independent Data Monitoring Committee (IDMC) initial discussions  *MoHS* |  |  | **X** |  |  |  |  |  |  |  |  |  |  |  | |  |  | | | |
| Questionnaire development & completion  *WHO/US-CDC* | **X** |  |  |  |  |  |  |  |  |  |  |  |  |  | |  |  | | | |
|  | **2015** | | | **2016** | | | | | | | | | | | | |  | | | |
|  | *Summer* | *Fall* | *December* | *January* | *February* | *March* | *April* | *May* | *June* | *July* | *August* | *September* | *October* | *November* | *December* | |  | | | |
| Training on participant recruitment  *MoHS/WHO/US-CDC* | **X** |  |  |  |  |  |  |  |  |  |  |  |  |  |  | |  | | | |
| Training on informed consent process  *MoHS/WHO/US-CDC* | **X** |  |  |  |  |  |  |  |  |  |  |  |  |  |  | |  | | | |
| Training on administering questionnaire  *MoHS/WHO/US-CDC* | **X** |  |  |  |  |  |  |  |  |  |  |  |  |  |  | |  | | | |
| Recruitment for pilot | **X** |  |  |  |  |  |  |  |  |  |  |  |  |  |  | |  | | | |
| Data collection for pilot | **X** | **X** | **X** | **X** | **X** | **X** | **X** | **X** | **X** | **X** | **X**  ***Last projected 6 month follow up visit*** |  |  |  |  | |  | | | |
|  | **2015** | | | **2016** | | | | | | | | | | | | |  |  |  | |
|  | *Summer* | *Fall* | *December* | *January* | *February* | *March* | *April* | *May* | *June* | *July* | *August* | *September* | *October* | *November* | *December* | |  | | | |
| Data analysis for pilot | **X** | **X** | **X** | **X** | **X** | **X** | **X** | **X** | **X** | **X** | **X** | **X** |  |  |  | |  | | | |
| Laboratory analysis for pilot  *US-CDC, China-CDC* | **X** | **X** | **X** | **X** | **X** | **X** | **X** | **X** | **X** | **X** | **X** | **X** |  |  |  | |  | | | |
| Recruitment for full study |  | **X** | **X** | **X** |  |  |  |  |  |  |  |  |  |  |  | |  | | | |
| Data collection for full study |  | **X** | **X** | **X** | **X** | **X** | **X** | **X** | **X** | **X** | **X** | **X** | **X** | **X** | **X**  ***Last projected 6 month follow up visit*** | |  | | | |
| Laboratory analysis  *China-CDC, US-CDC* |  | **X** | **X** | **X** | **X** | **X** | **X** | **X** | **X** | **X** | **X** | **X** | **X** | **X** | **X** | |  | | | |
| Writing interim project report  *WHO/US-CDC* |  |  | **X** | **X** |  |  |  |  |  |  |  |  |  |  |  | |  | | | |
|  | **2015** | | | **2016** | | | | | | | | | | | | |  |  |  |  |
|  | *Summer* | *Fall* | *December* | *January* | *February* | *March* | *April* | *May* | *June* | *July* | *August* | *September* | *October* | *November* | *December* | |  | | | |
| IDMC interim review |  |  |  | **X** |  |  |  |  |  |  |  |  |  |  |  | |  | | | |
| Writing final project reports  *MoHS / WHO/US-CDC* |  |  |  |  |  |  |  |  |  |  |  |  | **X** | **X** |  | |  | | | |
| IDMC final review |  |  |  |  |  |  |  |  |  |  |  |  | **X** |  |  | |  | | | |
| Final project review process  *MoHS* |  |  |  |  |  |  |  |  |  |  |  |  | **X** | **X** |  | |  | | | |
| Dissemination of project outcomes  *MoHS / WHO/US-CDC* |  |  |  |  |  |  |  |  |  |  |  |  |  |  | **X** | |  | | | |

**9. Main problems anticipated and proposed solutions**

Main challenges to the success of the project include willingness of participants to provide biological specimens from sensitive locations and involving masturbation for men, and to share intimate information on sexual behavior. Proposed solutions are to ensure trained interviewers and medical staff of appropriate genders and cultural backgrounds, who are used to discussing sexual behavior, and to take bio-samples of sensitive character. Also to secure an environment where the interviewee will be secure the procedures and information is kept confidential. The ability to provide quick turnaround of specimen processing and analysis for timely report back to participants, especially in the breast milk module will also be a challenge.

**10. Applicability of results**

The results will be of great relevance to provide evidence-based information to circumvent EBOV transmission from patients recovered from EVD. They will inform the development of recommendations used in the current epidemic setting, as well as in future situations with resurgence of EBOV. The inclusion of PLHIVs in the study will allow documentation related to co-infection and potential changes to HIV programming during EVD outbreaks.

**11. Gender considerations**

Both male and female participants will be enrolled in this study.

**11.1 Describe how women and men are affected by the public health need that the study addresses, and whether this is a need expressed or felt by women and /or men**

Both men and women are significantly affected by the EBOV epidemic. Women are likely more vulnerable to the risk of sexual transmission of EBOV, given both their biological vulnerability related to the vaginal mucosa and structural gender inequities that give women limited possibilities to negotiate condom use. Men have been victims of law-enforcement actions in relation to suspicions of sexual transmission of EBOV.

In addition, survivors are reporting the need for reassurance of their status with regard to potentially infectious EBOV and the importance for them to know if they may return to their normal sexual practices.

Additionally, women are also more vulnerable to HIV infection in addition to the aforementioned vulnerability to sexual transmission of EBOV, therefore the inclusion of PLHIV will contribute to the discussion on the differences in public health need between genders.

**11.2 Explain how the research contributes to identifying and/or reducing inequities between women and men in sexual and reproductive health and health care**

The research aims at verifying the persistence of EBOV among both women and men, and to assess associations to suspected sexual transmission of EBOV, and hence aims at providing key-information to both women and men. In addition evidence-based strategies will inform human rights based response to controlling transmission that does not include imprisonment or isolation.

**11.3 Describe measures taken to facilitate the individual participation of women or men in the research process in light of their different life situations**

Recruitment will happen at ETUs and from survivor registries, with enrollment targets that include both men and women. Actions will be taken to ensure both women and men may be interviewed privately and in an environment where they are at ease. Interviewers of the same sex as the participant will secure a place and time of interview that is private and does not interfere with other pertinent activities. Special precautions will be taken to protect survivors where secondary cases occur, in order to avoid a response that is not evidence-and rights-based.

**11.4 Describe measures taken to ensure that community involvement is inclusive**

Explorative focus group discussions with key stakeholders in the community are being proposed. The discussions will focus on community involvement and a needs-based approach. PLHIV support groups located in the community level will also be engaged in identifying PLHIV EVD survivors to approach for recruitment to the study.

**11.5 Describe the sex composition of the research team, and their duties and responsibilities in the proposed research**

The research team comprises both men and women (see above).

**12. Ethical considerations**

**12.1 Study population, recruitment strategy and informed consent process**

• Recruitment to the cohort will take place at the ETUs at the time of discharge or later on via survival registers or clinics; informed consent will be explained by trained research staff at the time of recruitment at the study site.

• All adult (18 years or above) men or women, consecutively discharged or in convalescent care, with a confirmed recovered EVD should hence be informed about the study and invited to participate, providing informed consent in writing (Appendix 1, see Ethics section below) (along Main study).

• Due to the confidential nature of HIV status, PLHIV support groups will be engaged in order to identify and recruit PLHIV EVD survivors. HIV Counselors will also be engaged to encourage newly diagnosed PLHIVs who are also EVD survivors.

**12.2 Perceived risks and benefits of the study, both at the individual and community levels**

The project is dependent on willingness from participants to share intimate information on sexual behavior, and to provide biological specimens. For the individual, there is a risk of the intimate interview and sampling procedure creating anxiety for the interviewee and for his/her family members. Specimen collection may cause discomfort, pain, or local infection (Any invasive specimen collection can have side effects that should be detailed here. In particular, vaginal and rectal swabs can rarely cause local trauma or infection that is usually minor. Study participants receiving their results may cause psychological and social stress, especially when results are of unknown clinical utility and may be subject to misinterpretation. At the community level, there is a risk of stigma or discrimination enacted towards study participants. For PLHIVs, there is a risk of unconsented disclosure of HIV status.

Benefits of the study include possibilities for participants to have information on any viral persistence in body fluids, for the community to get accurate recommendations on how to stop transmission chains. For the HIV sector, a benefit would be an evidence-based understanding of whether HIV affects EVD survivors differently and result in potential changes in programming during EVD outbreaks. Participants will receive a monetary incentive at each study visit, as well as condoms, counseling, and linkages to survivor resources.

**12.3 Safeguards to protect any recognized vulnerability of the study participants**

Only staff and interviewers trained in specimen collection and discussion of sensitive sexual behavior will be used in the proposed study. Also, to secure an environment where the interviewee will be secure the information is kept confidential. Personally identifying information (e.g., names) is not transmitted with specimens. Confidentiality of PLHIVs will be safeguarded by only using HIV support groups and HIV counselors (therefore, people

already known to the PLHIV) to communicate with them prior to enrolment of the study. The counselling script for women includes questions to recognize intimate partner violence and offer referral if the participant is experiencing this.

**12.4 Reimbursement or compensation to study participants**

Participants will receive compensation at the initial visit and again at every subsequent

follow-up visit. Determination of the compensation amount follows: 50,000 Leones will be for attending a study visit, an additional 50,000 Leones will cover transportation costs of the participant, and 20,000 Leones will cover the costs of a meal. The total amount that a participant will receive at each visit will be 120,000 Leones. (Participants may choose to spend their compensation as they wish.) At every visit, 5 weeks’ worth of condoms (approximately 35 condoms) will be given to the participant, as well as formula as needed to breast feeding women. Participants will receive compensation for attending a study visit even if they do not complete a study visit (e.g. incomplete interview or specimen collection). Participants who do not complete multiple study visits may be deemed ineligible from participating further.

**12.5 Access to treatment or counselling for conditions either identified during screening of potential participants or resulting from the study intervention**

Participants will be offered timely risk reduction counseling on prevention of transmission of EBOV by trained counselors. In addition, all participants in need of specific health services (including physical or mental health, participants experiencing intimate partner violence, or breastfeeding women whose breast milk tests positive and need alternative sources of nutrition for their babies) will be referred to the appropriate clinic/hospital for care and management. HIV positive participants will be referred to the national program including HIV counseling and treatment services.

**12.6 Responsiveness of the project to community needs and priorities**

This study was developed to address community concerns about the potential for transmitting EBOV via sexual and other close contact. The MoHS worked closely with EVD survivors with WHO and US-CDC providing technical assistance.

The cohort study will contribute to evidence- and human rights based response to the Ebola epidemic and suspicions of transmission through body fluids by survivors. It will counteract human rights violations such as law-enforcing actions against patients suspected to transmit.

**12.7 Deception**

See above.

**13. Forms required**

See operations manual.

**14. Plans for dissemination and use of project results**

Results and analysis (both interim and final) will be used to update and refine relevant counseling messages and recommendations from WHO, US-CDC. Potential products include scientific abstracts and manuscripts, presentations, guidance documents, and others. A memorandum of understanding (MOU) and a material transfer agreement (MTA) will be developed in advance of the study between MoHS, WHO, US-CDC and Chinese-CDC in order to ensure collaboration and appropriate recognition of all contributing co-authors. All parties (MoHS, WHO, US-CDC, Chinese-CDC) will share ownership of all data in the study. Publication policy will follow international guidelines for authorship applying Vancouver criteria, and all publications will be shared and reviewed by the three parties before submission for publication. Local collaborations areinvolved with the UNAIDS country office, with MSWGCA and local survivors’ organisations (SLAES). These local partners will support the study process in different respects, during the data collection process and/or in relation to specific needs of study participants. Local partners will be offered co-authorship in relation to fulfilling the Vancouver criteria for authorship.

**Dissemination of project results to study participants:**

As many of the study participants have low literacy rates, distribution of written summaries of study results is not appropriate. Throughout the pilot and continuing into the main study, results from the study are, and will continue to be, disseminated to survivors principally via the Sierra Leone Association of Ebola Survivors (SLAES). The study team has a constructive working relationship with SLAES; and their executive board is briefed in layman’s language, in real-time, via presentations conducted by senior study staff at the study site. These meetings also involve lengthy question and answer sessions. The study team also regularly attends SLAES meetings to answer any questions arising with regards to the study. This function is primarily conducted by the studies community liaison officers, who are themselves survivors and members of SLAES. Executive members of SLAES (from multiple districts of Sierra Leone) have also participated in a media workshop (August 2015), where the study team, in collaboration with a media NGO (PCI Media Impact), developed messaging around the pilot study baseline RT-PCR results.

**Briefing the Government of Sierra Leone and Partner organizations in Real-time:**

The study team also briefs the Government of Sierra Leone and partner organizations regularly. Presentations of study results are given in real-time at high level MOHS briefings and to the National Ebola Response Centre (NERC). The NERC is led by the Ministry of Defense, with key partner organizations in the Ebola response in attendance. In Sierra Leone, the Ebola response effort has been managed by Government led pillars.

The Ministries of Health and the WHO country offices in Guinea and Liberia have been informed of the results from the pilot study, in real-time, via periodic presentations.

**Communications surrounding publications:**

The communication around the publication of the pilot baseline RT-PCR data (Deen et al., 2015) was carefully managed by dedicated communications experts from the MOHS, WHO and US-CDC. Together with the studies technical leads, these communications experts crafted a press release, headed by the MOHS, which was read out on local radio multiple times during the day that the publication was released. Radio is one of the most effective means of disseminating community wide information in Sierra Leone. This is the format that will be followed for any other publication from the study.

The SLAES executive board was made aware of the exact timing of the publication and multiple presentations on the results and the updated interim recommendations on sexual practices of Ebola survivors were given by the study team to the following pillars and sub-committees; communications pillar, social mobilization pillar, health promotions pillar; and the messaging sub-committee. This information has also been presented to a WHO community engagement workshop with meeting participants covering all districts of Sierra Leone.

**15 References**

Bausch DG, Towner JS, Dowell SF, Kaducu F, Lukwiya M, Sanchez A, [Nichol ST](http://www.ncbi.nlm.nih.gov/pubmed/?term=Nichol ST%5BAuthor%5D&amp;cauthor=true&amp;cauthor_uid=17940942), [Ksiazek T](http://www.ncbi.nlm.nih.gov/pubmed/?term=Ksiazek TG%5BAuthor%5D&amp;cauthor=true&amp;cauthor_uid=17940942)G[,](http://www.ncbi.nlm.nih.gov/pubmed/?term=Rollin PE%5BAuthor%5D&amp;cauthor=true&amp;cauthor_uid=17940942) Rollin PE. Assessment of the risk of Ebola virus transmission from bodily fluids and fomites. *J Infect Dis*. 2007;196 Suppl 2:S142-7.

Centers for Disease Control and Prevention (CDC). CDC Ebola Response Update, March 8,

2015. Accessed March 10, 2015. Available from:

<http://www.cdc.gov/vhf/ebola/outbreaks/2014-west-africa>

Centers for Disease Control and Prevention (CDC). Supporting West African Ebola Survivors. Accessed March 10, 2015. Available from: <http://www.cdc.gov/media/releases/2014/p1212-ebola-survivors.html>

Chertow DS, Kleine C, Edwards JK, Scaini R, Giuliani R, Sprecher A. Ebola virus disease in

West Africa--clinical manifestations and management. *N Engl J Med*. 2014 Nov

27;371(22):2054-7.

Dowell SF, Mukunu R, Ksiazek TG, Khan AS, Rollin PE, Peters CJ, Commission de Lutte contre les Epidémies à Kikwit. Transmission of Ebola hemorrhagic fever: a study of risk factors in family members, Kikwit, Democratic Republic of the Congo, 1995. *J Infect Dis*.

1999 Feb;179 Suppl 1:S87-91.

Emond RT, Evans B, Bowen ET, Lloyd G. A case of Ebola virus infection. *British Medical*

*Journal*. 1977;2(6086):541-4.

Francesconi P, [Yoti Z](http://www.ncbi.nlm.nih.gov/pubmed/?term=Yoti Z%5BAuthor%5D&amp;cauthor=true&amp;cauthor_uid=14718087)[,](http://www.ncbi.nlm.nih.gov/pubmed/?term=Declich S%5BAuthor%5D&amp;cauthor=true&amp;cauthor_uid=14718087) Declich S[,](http://www.ncbi.nlm.nih.gov/pubmed/?term=Onek PA%5BAuthor%5D&amp;cauthor=true&amp;cauthor_uid=14718087) Onek PA, [Fabiani M,](http://www.ncbi.nlm.nih.gov/pubmed/?term=Fabiani M%5BAuthor%5D&amp;cauthor=true&amp;cauthor_uid=14718087) [O](http://www.ncbi.nlm.nih.gov/pubmed/?term=Olango J%5BAuthor%5D&amp;cauthor=true&amp;cauthor_uid=14718087)lango [J,](http://www.ncbi.nlm.nih.gov/pubmed/?term=Andraghetti R%5BAuthor%5D&amp;cauthor=true&amp;cauthor_uid=14718087) Andraghetti R, [Rollin PE](http://www.ncbi.nlm.nih.gov/pubmed/?term=Rollin PE%5BAuthor%5D&amp;cauthor=true&amp;cauthor_uid=14718087), [Opira C](http://www.ncbi.nlm.nih.gov/pubmed/?term=Opira C%5BAuthor%5D&amp;cauthor=true&amp;cauthor_uid=14718087)[,](http://www.ncbi.nlm.nih.gov/pubmed/?term=Greco D%5BAuthor%5D&amp;cauthor=true&amp;cauthor_uid=14718087) Greco D[,](http://www.ncbi.nlm.nih.gov/pubmed/?term=Salmaso S%5BAuthor%5D&amp;cauthor=true&amp;cauthor_uid=14718087) Salmaso S. Ebola hemorrhagic fever transmission and risk factors of contacts, Uganda. *Emerg Infect Dis* 2003;9:1430-1437.

Kortepeter MG, Bausch DG, Bray M. Basic clinical and laboratory features of filoviral hemorrhagic fever. *J Infect Dis*. 2011 Nov;204 Suppl 3:S810-6.

Kreuels B, [Wichmann D](http://www.ncbi.nlm.nih.gov/pubmed/?term=Wichmann D%5BAuthor%5D&amp;cauthor=true&amp;cauthor_uid=25337633), [Emmerich P](http://www.ncbi.nlm.nih.gov/pubmed/?term=Emmerich P%5BAuthor%5D&amp;cauthor=true&amp;cauthor_uid=25337633), [Schmidt-Chanasit J](http://www.ncbi.nlm.nih.gov/pubmed/?term=Schmidt-Chanasit J%5BAuthor%5D&amp;cauthor=true&amp;cauthor_uid=25337633), [de Heer G](http://www.ncbi.nlm.nih.gov/pubmed/?term=de Heer G%5BAuthor%5D&amp;cauthor=true&amp;cauthor_uid=25337633), [Kluge S](http://www.ncbi.nlm.nih.gov/pubmed/?term=Kluge S%5BAuthor%5D&amp;cauthor=true&amp;cauthor_uid=25337633), [Sow A](http://www.ncbi.nlm.nih.gov/pubmed/?term=Sow A%5BAuthor%5D&amp;cauthor=true&amp;cauthor_uid=25337633), [Renné T](http://www.ncbi.nlm.nih.gov/pubmed/?term=Renné T%5BAuthor%5D&amp;cauthor=true&amp;cauthor_uid=25337633)[,](http://www.ncbi.nlm.nih.gov/pubmed/?term=Günther S%5BAuthor%5D&amp;cauthor=true&amp;cauthor_uid=25337633) Günther S, [Lohse AW](http://www.ncbi.nlm.nih.gov/pubmed/?term=Lohse AW%5BAuthor%5D&amp;cauthor=true&amp;cauthor_uid=25337633)[,](http://www.ncbi.nlm.nih.gov/pubmed/?term=Addo MM%5BAuthor%5D&amp;cauthor=true&amp;cauthor_uid=25337633) Addo MM, [S](http://www.ncbi.nlm.nih.gov/pubmed/?term=Schmiedel S%5BAuthor%5D&amp;cauthor=true&amp;cauthor_uid=25337633)chmiedel S. A Case of Severe Ebola Virus Infection Complicated by Gram-Negative Septicemia. *N Engl J Med*. 2014 Dec

18;371(25):2394-401.

Ksiazek TG, Rollin PE, Williams AJ, Bressler DS, Martin ML, Swanepoel R, Burt FJ, Leman PA, Khan AS, Rowe AK, Mukunu R, Sanchez A, Peters CJ. Clinical virology of Ebola hemorrhagic fever (EHF): virus, virus antigen, and IgG and IgM antibody findings among EHF patients in Kikwit, Democratic Republic of the Congo, 1995. *J Infect Dis*. 1999 Feb;179

Suppl 1:S177-87.

Mackay IM, Arden KE. Ebola virus in the semen of convalescent men. *Lancet Infect Dis*.

2015 Feb;15(2):149-50.

Martini GA, Schmidt HA. [Spermatogenic transmission of the "Marburg virus". (Causes of

"Marburg simian disease")]. *Klinische Wochenschrift*. Apr 1 1968;46(7):398-400. German.

[Moreau M,](http://www.ncbi.nlm.nih.gov/pubmed/?term=Moreau M%5BAuthor%5D&amp;cauthor=true&amp;cauthor_uid=25635320) [S](http://www.ncbi.nlm.nih.gov/pubmed/?term=Spencer C%5BAuthor%5D&amp;cauthor=true&amp;cauthor_uid=25635320)pencer C[,](http://www.ncbi.nlm.nih.gov/pubmed/?term=Gozalbes J%5BAuthor%5D&amp;cauthor=true&amp;cauthor_uid=25635320) Gozalbes J, [Colebunders R](http://www.ncbi.nlm.nih.gov/pubmed/?term=Colebunders R%5BAuthor%5D&amp;cauthor=true&amp;cauthor_uid=25635320), [Lefevre A](http://www.ncbi.nlm.nih.gov/pubmed/?term=Lefevre A%5BAuthor%5D&amp;cauthor=true&amp;cauthor_uid=25635320)[,](http://www.ncbi.nlm.nih.gov/pubmed/?term=Gryseels S%5BAuthor%5D&amp;cauthor=true&amp;cauthor_uid=25635320) Gryseels S, [Borremans B](http://www.ncbi.nlm.nih.gov/pubmed/?term=Borremans B%5BAuthor%5D&amp;cauthor=true&amp;cauthor_uid=25635320), [Gunther S](http://www.ncbi.nlm.nih.gov/pubmed/?term=Gunther S%5BAuthor%5D&amp;cauthor=true&amp;cauthor_uid=25635320), [Becker D](http://www.ncbi.nlm.nih.gov/pubmed/?term=Becker D%5BAuthor%5D&amp;cauthor=true&amp;cauthor_uid=25635320), [Bore J](http://www.ncbi.nlm.nih.gov/pubmed/?term=Bore J%5BAuthor%5D&amp;cauthor=true&amp;cauthor_uid=25635320), [Koundouno F](http://www.ncbi.nlm.nih.gov/pubmed/?term=Koundouno F%5BAuthor%5D&amp;cauthor=true&amp;cauthor_uid=25635320), [Di Caro A](http://www.ncbi.nlm.nih.gov/pubmed/?term=Di Caro A%5BAuthor%5D&amp;cauthor=true&amp;cauthor_uid=25635320), [Wolfel R](http://www.ncbi.nlm.nih.gov/pubmed/?term=Wolfel R%5BAuthor%5D&amp;cauthor=true&amp;cauthor_uid=25635320)[,](http://www.ncbi.nlm.nih.gov/pubmed/?term=Decroo T%5BAuthor%5D&amp;cauthor=true&amp;cauthor_uid=25635320) Decroo T, [Van Herp M](http://www.ncbi.nlm.nih.gov/pubmed/?term=Van Herp M%5BAuthor%5D&amp;cauthor=true&amp;cauthor_uid=25635320), [Peetermans L](http://www.ncbi.nlm.nih.gov/pubmed/?term=Peetermans L%5BAuthor%5D&amp;cauthor=true&amp;cauthor_uid=25635320), [Camara](http://www.ncbi.nlm.nih.gov/pubmed/?term=Camara A%5BAuthor%5D&amp;cauthor=true&amp;cauthor_uid=25635320) A. Lactating mothers infected with Ebola virus: EBOV RT-PCR of blood only may be insufficient. *Euro Surveill*. 2015 Jan 22;20(3). [pii: 21017](http://www.eurosurveillance.org/ViewArticle.aspx?ArticleId=21017).

O'Dempsey T, Khan SH, Bausch DG. Rethinking the Discharge Policy for Ebola

Convalescents in an Accelerating Epidemic. *Am J Trop Med Hyg*. 2015 Feb 4;92(2):238-9.

Richards GA, [Murphy S](http://www.ncbi.nlm.nih.gov/pubmed/?term=Murphy S%5BAuthor%5D&amp;cauthor=true&amp;cauthor_uid=10667531), [Jobson R](http://www.ncbi.nlm.nih.gov/pubmed/?term=Jobson R%5BAuthor%5D&amp;cauthor=true&amp;cauthor_uid=10667531)[,](http://www.ncbi.nlm.nih.gov/pubmed/?term=Mer M%5BAuthor%5D&amp;cauthor=true&amp;cauthor_uid=10667531) Mer M, [Zinman C](http://www.ncbi.nlm.nih.gov/pubmed/?term=Zinman C%5BAuthor%5D&amp;cauthor=true&amp;cauthor_uid=10667531)[,](http://www.ncbi.nlm.nih.gov/pubmed/?term=Taylor R%5BAuthor%5D&amp;cauthor=true&amp;cauthor_uid=10667531) Taylor R, [Swanepoe](http://www.ncbi.nlm.nih.gov/pubmed/?term=Swanepoel R%5BAuthor%5D&amp;cauthor=true&amp;cauthor_uid=10667531)l R, [Duse A](http://www.ncbi.nlm.nih.gov/pubmed/?term=Duse A%5BAuthor%5D&amp;cauthor=true&amp;cauthor_uid=10667531), [Sharp G](http://www.ncbi.nlm.nih.gov/pubmed/?term=Sharp G%5BAuthor%5D&amp;cauthor=true&amp;cauthor_uid=10667531), [De La Rey IC](http://www.ncbi.nlm.nih.gov/pubmed/?term=De La Rey IC%5BAuthor%5D&amp;cauthor=true&amp;cauthor_uid=10667531)[,](http://www.ncbi.nlm.nih.gov/pubmed/?term=Kassianides C%5BAuthor%5D&amp;cauthor=true&amp;cauthor_uid=10667531) Kassianides C. Unexpected Ebola virus in a tertiary setting: clinical and epidemiologic aspects. *Critical Care Medicine*. Jan 2000;28(1):240-244.

Rodriguez LL, De Roo A, Guimard Y, Trappier SG, Sanchez A, Bressler D, Williams AJ, Rowe AK, Bertolli J, Khan AS, Ksiazek TG, Peters CJ, Nichol ST. Persistence and genetic stability of Ebola virus during the outbreak in Kikwit, Democratic Republic of the Congo,

1995. *J Infect Dis*. 1999;179 Suppl 1:S170-6.

Rogstad KE, Tunbridge A. Ebola virus as a sexually transmitted infection. *Curr Opin Infect*

*Dis.* 2015 Feb;28(1):83-5.

Rowe AK, Bertolli J, Khan AS, Mukunu R, Muyembe-Tamfum JJ, Bressler D, Williams AJ, Peters CJ, Rodriguez L, Feldmann H, Nichol ST, Rollin PE, Ksiazek TG. Clinical, virologic, and immunologic follow-up of convalescent Ebola hemorrhagic fever patients and their household contacts, Kikwit, Democratic Republic of the Congo. Commission de Lutte contre les Epidemies a Kikwit. The Journal of infectious diseases. 1999;179 Suppl 1:S28-35.

Sonnenberg P, Field N. Sexual and Mother-to-Child Transmission of Ebola Virus in the

Postconvalescent Period. *Clin Infect Dis*. 2015 Mar 15;60(6):974-5.

WHO Ebola Response Team. Ebola virus disease in West Africa--the first 9 months of the epidemic and forward projections. *N Engl J Med*. 2014 Oct 16;371(16):1481-95.

World Health Organization (WHO). Ebola Report for week of February 15, 2015.

**Survivor Study Tools are independent documents:**

Annex 1 Questionnaires

Annex 2 Counselling scripts

Operations Manual (included all the SOPs)

**Appendix 1a Informed Consent Form for study participants**

**Consent form for participants in the study of Ebola virus in body fluids from survivors**

**This Informed Consent Form has two parts:**

**• Information Sheet (to share information about the research with you)**

**• Certificate of Consent (for your signature or mark if you agree to take part) You will be given a copy of the full Informed Consent Form.**

**PART I: Information Sheet**

**Introduction**

Hello, I am a health care worker with the Ministry of Defense (MoD). The Ministry of Health and Sanitation (MoHS) together with the World Health Organization (WHO), the U.S. Centers for Disease Control and Prevention (US-CDC) and Chinese Centers for Disease Control and Prevention (C-CDC) are conducting a research study and we would like to talk to you about participating. We are looking for participants who are adults over the age of 18 years who have survived Ebola Virus Disease. Can I give you more information?

This goal of this research study is to investigate how long the virus can be found in different body fluids of survivors, other than blood. The main investigator for this research study is Dr. Deen from the MOHS.

I would like to give you information about the research study and invite you to participate if you are eligible. It is your decision whether you want to participate or not. Please feel free to ask any questions of me, the main investigator, or anyone else at any time so that you can feel comfortable making a decision to participate in this research study or not.

**Purpose of the research**

There is a lot about the Ebola virus disease that we do not know or understand, and we have been learning from the epidemic in West Africa. We know that Ebola is a contagious disease that is most commonly passed from person to person by direct contact with body fluids like vomit, stool, or blood from a person who is sick with Ebola. In the Ebola Treatment Center, we check blood tests to make sure the virus is no longer present in the blood before a survivor is discharged home. However, the Ebola virus may still be present in small amounts in other body fluids of survivors. For example, before this epidemic in West Africa, we knew that live Ebola virus had been found in male semen for up to 3 months after recovery. From the pilot study that we have conducted (in Freetown, 2015) we now know that for some people pieces of the virus can remain in semen for many months longer than this (9 months or more). This research study will investigate how long the Ebola virus can be found in body fluids from survivors including semen (if you are a man), vaginal fluid or menstrual blood (if you are a woman), breast milk (if you are a lactating woman) as well as saliva, urine, stool, sweat, and tears from all participants who agree to provide these samples.

If you are an Ebola survivor over the age of 18 years, your participation in this study is important for our research to understand how long the Ebola virus can be found in body fluids other than blood.

**Type of Research Intervention**

We are inviting Ebola survivors to undergo testing for the Ebola virus on samples of their body fluids in order to learn how long it may take the virus or pieces of the virus to leave these different types of body fluids. We are not studying a new type of medicine or a new type of treatment as part of this research study and therefore the research does not involve risks that are not known in advance. However, the results of the presence of the virus in a survivor’s body fluids may generate stigma and discrimination and this is addressed under the paragraph “Risk and benefit”.

**Participant selection**

For this study, we invite adult men and women at least 18 years old who have recovered from Ebola virus disease. Upon volunteering for this study we will ask you to present your Ebola discharge/survivors’ certificate.

- ***Example of question to elucidate understanding:*** *Do you understand what the study is about?*

**Voluntary Participation**

Your participation in this research study is entirely voluntary. It is your choice whether to participate or not. Whether you choose to participate or not, you can continue to receive all the same services offered by the MoHS for survivors, and nothing concerning your medical care will change. You may change your mind and stop participating at any time, even if you had agreed to participate earlier. If you wish to withdraw from the study you will still have the opportunity to speak further about the test results and measures that can reduce the risk of transmitting Ebola to other persons. If you wish to withdraw from the study you will still have the opportunity to speak further about the test results and measures that can reduce the risk of transmitting Ebola to other persons.

➢ ***Examples of question to elucidate understanding:*** *Do you know that you do not have to take part in this research study, if you do not wish to? Do you have any questions?*

**Overview of Study Procedures**

Study participants will be invited to attend several study visits at the study clinic. A member of the study team will call you to remind you to attend upcoming visits. At each visit a trained counselor or nurse will ask a set of interview questions in a private setting. After the interview, we will request that you submit samples of certain body fluids depending on if you are a man or women or a lactating women (for example: semen, vaginal swab for vaginal secretions or menstrual blood, saliva, urine, rectal swab for stool, sweat, tears or breast milk). Everyone will be asked for a blood test to assess antibodies to the Ebola virus. All samples will be sent to the Chinese-CDC laboratory in Jui, Sierra Leone, and all samples (except the blood sample) will be tested to see if pieces of the Ebola virus are present. Each study visit will last more than an hour, and visits will be two weeks apart for all participants.

An optional HIV test and counseling will be offered to all participants. If you chose to be tested, the results will be kept confidential and will be shared with you. If you test HIV positive, your will be referred to HIV services provided by the MoHS. If you are someone alreadyl living with HIV, a confirmatory test will be offered.

Pregnancy testing will be offered to all women who would like to receive one, and the results will be kept confidential. If you are pregnant we will put you in contact with antenatal care as you wish.

If you are pregnant, we would like to get information about your delivery. We will ask your healthcare provider to give us this information.

For lactating women test results will be provided within 3 days, and if the test is positive we will collect another sample on the same day.

For menstruating women, we will ask you to provide a specimen on the first day of your period, and we will try to have a second specimen collected during the same menstrual period.

In addition to the body fluids testing, we would also like to access your records from the laboratory and ETU clinic from the time you were treated for Ebola. Access to the information will permit to check the information we have from the questionnaires you have completed with the nurse, to ensure we have the right information.

**To sign the informed consent will imply that you grant us access to your records from the laboratory and ETU clinic from the time you were treated for Ebola**

**Read to participants in the HIV positive cohort only:** Although you have already been tested and know your status, a confirmatory HIV test will be requested. If you chose to be tested, the results will be kept confidential and will be shared with you. If you test HIV positive, your will be referred to HIV services provided by the MoHS.

You will be given all of your Ebola test results and the results will be explained to you. When you have two negative tests in a row from all the body fluids tested, we will stop collecting body fluids. These tests for Ebola virus pieces are very reliable, however it may be possible that virus could still be present in the body but not able to be found in body fluids. There have been a very small number of survivors who became seriously ill after they recovered, and so we would like to ensure that you are well even after finishing the study.

If you agree, you will be invited for a new visit at 3 and 6 months after your second negative sample. We will assess your health status by asking you questions about your health and will test again your body fluids again at these time points. In case one of your body fluids tests positive at one of these two visits, then the same protocol as before when enrolled in the study will be observed, and we will follow you-up until you have two consecutive negative test results in a row for all body fluids.

**Do you agree to have a 3 and 6 month visit? Yes No**

**At any time, we recommend you contact us if you have fever, fatigue, or feel unwell.**

**Description of Participation**

At the study visits, you will be asked a set of questions about the time you were sick with Ebola, some general questions about your health, as well as personal questions about your sexual behavior after you recovered from your illness and from the time of your last visit. The interviews will be done in private by a trained health care worker and all of your answers will be kept confidential. We will check your clinical and laboratory records from this time to get additional information about the time you were acutely sick with Ebola. You can decline to answer certain questions if you do not feel comfortable or simply choose not to answer. If you decide not to answer certain questions you can still participate in the study.

In order to check whether the Ebola virus is still present in certain body fluids, we will test samples from your body that you agree to provide. Sample collection will be done in a private place and the samples can either be collected by yourself after we give detailed instructions, or a gender appropriate health care worker will assist you. For samples that are not intimate, they will be taken in the presence of a trained health care worker, who will assist you.

For example, with your permission, a health care worker will collect a small amount of blood from a vein in your arm. A trained health care worker may collect sweat from anywhere that you are visibly sweating (for example your back, forehead or top lip), saliva from your mouth, and tears from your eyes. The other specimens you can collect yourself in private, or if you wish the health care worker can help you. For the urine test we will ask you to pass urine into a special cup. For the rectal swab, we will give you a soft cotton swab to check your anus. The swabbing is not usually considered painful, but may feel uncomfortable.

**Read to female participants only**: If you are a woman, we will give you another soft cotton swab to check your vagina yourself or you can have a nurse assist you. The same procedure will be used for collecting menstrual blood. If you are breastfeeding the healthcare worker will help you to collect breast milk, or you can do this by yourself if you wish.

**Read to male participants only**: For the semen collection, we will ask you to spend time alone in a private room to masturbate and provide semen in a special cup

All of these samples will be sent to the Chinese-CDC laboratory, Jui, in Sierra Leone for initial testing for the Ebola virus and the test results will be shared with you. This tests looks for pieces of the Ebola Virus.

If the test is positive that body fluid sample (except for semen) will be sent to the US-CDC in the United States for further testing to find out if the virus in the sample is alive, and may be able to infect others. It could take several months to get these results from US-CDC but the results will be shared with you.

We may also do additional testing at the Chinese-CDC lab in Jui to describe the virus.

The samples obtained will only be used for the here described Ebola testing, and will be safely and respectfully destroyed after this research is completed. The Ebola virus grown in the laboratory may be saved and used for future scientific studies should you agree. The scientific use of any virus kept for additional studies, would be decided upon by the group of study responsible persons from the Ministry of Health and Sanitation, Sierra Leone, the WHO, US-CDC and Chinese-CDC.

You can decide if you allow for future study of the virus that might be produced from your samples or if you prefer that it is destroyed.

If you do not want the laboratory to keep any Ebola virus isolates that stem from your body fluids you may say so now, we will record it, and no virus will be stored.

**Agree for laboratory to store virus: yes no**

**(Circle appropriate**

**answer)**

During your first visit we will also offer you the possibility to get tested for HIV if you would like. The test involves a skin prick where we take a few drops of blood for analyses. If you test positive, immediately afterwards a second test will be done to confirm your status. The answer to the test will be given to you in the same confidential setting by the same health care worker/counsellor that you discuss with in relation to Ebola. You will get the answer to the HIV test the same day. Should your test be positive the counsellor/health worker will explain to you where to find further care and support, including treatment if needed.

**Participation**

Each study visit should last about an hour, but might be shorter or longer depending on your needs. It is possible that some people will need study visits every two weeks for several months, and other people will need only three study visits total.

At each visit you will be seen by a nurse and a laboratory technician who will help with specimen collection. When needed, or if you have any particular questions, the study physician will come and meet with you.

You will be asked to return for another study visit initially after 2 weeks, then after 2-4 weeks (or for lactating or menstruating women, weekly) until you have two negative tests in a row from all of your body fluids.

- ***Examples of question to elucidate understanding:*** *Do you have any other questions? Do you want me to go through the procedures again?*

**Side Effects**

This study does not provide any medication or experimental treatments that would cause side effects.

**Risks and Benefits**

Some people may feel embarrassed discussing private topics during the interview, but the trained male and female nurses and counselors will keep all information confidential. Specimen collection may be uncomfortable and might cause pain or local infection. If this occurs, a study physician will be available to see you. In the unlikely event that you need medical treatment, your will be referred to the nearest MoHS survivor clinic or medical facility, without any charge. Receiving results of Ebola testing might be stressful to you. There is also a risk of possible stigma or discrimination against you from others in your community

Benefits of the study include the opportunity for you to receive the results of Ebola tests on your body fluids and counseling on the meaning of the test results as well as information on how to prevent transmitting the virus to other people. It is your choice whether or not to receive the test results. Either way, the results will help inform our understanding of the Ebola virus and best ways to prevent Ebola from spreading in the future.

Participants will receive a monetary compensation of 120,000 Leones at each study visit, as well as condoms, counseling, and linkages to health services as needed. The 120,000

Leones will include coverage of the cost of a meal and for transport (estimated to be 70,000

Leones). They will receive a monetary compensation of 120,000 Leones at each study visit, as well as condoms given by the nurse at the end of each visit, and a receipt will be signed

by the participant.

**Incentives**

Participants will receive a monetary incentive of 120,000 Leones at each study visit, as well as condoms, counseling, and linkages to survivor resources.

- ***Examples of question to elucidate understanding:*** *Can you tell me about the risks and benefits of participating in this study?*

**Confidentiality**

Your study participation will be confidential. That means that your interview responses and Ebola test results will not be linked to your name or other personally identifying information. You will receive a study ID card with a number to identify you. You can answer the questions freely and all answers and results will be kept strictly confidential. Only the researchers will know what your study ID number is and we will lock that information up with a lock and key. We will not be sharing the identity of those participating in the research.

- ***Example of question to elucidate understanding:*** *Did you understand the procedures that we will be using to make sure that any information that we as researchers collect about you will remain confidential? Do you have any questions about them?*

**Dissemination**

The summary of this research will be shared with the scientific community in the form of scientific publications. In addition, the summary will be provided to the communities affected by the Ebola epidemic through educational campaigns provided by the MoHS. It is very important to provide the most accurate information about the Ebola virus and how long it may be found in body fluids of survivors in order to stop transmission of the virus.

**Right to Refuse or Withdraw**

You are not required to participate in this study and refusing or withdrawing will not affect you in any way, or your family or your access to clinic treatment. You will still have all the benefits that you would otherwise have in relation to needs of treatment at the clinic. You may stop participating in the research at any time that you wish without losing any of your rights as a patient or member of the community. For participants who wish to withdraw from the study you will still have the opportunity to speak further about the test results and measures that can reduce the risk of transmitting Ebola to other persons.

**Who to Contact**

You can always contact the main study investigator, Dr. Deen, Director of Clinical Studies at Connaught Hospital, Freetown – Tel: 076865597. In addition each participant will be given the name and contact telephone number of the study physician for the sites of enrollment and follow-up.

**This proposal has been reviewed and approved by an ethical review board linked to the Ministry of Health in Sierra Leone, which is a committee whose task it is to make sure that research participants are protected from harm. It has also been reviewed by the Ethics Review Committee of the World Health Organization (WHO), which is funding/sponsoring/ and supporting the study.**

Do you have any questions? Are you willing to participate in this research study?

**PART II: Certificate of Consent**

For those that decide to participate in this research study, all the specimen that have been taken will be safely and respectfully destroyed after this research is finished. You can decide if you allow scientists to keep any Ebola virus that might be produced from the samples you provide in a secure laboratory to study in the future.

I allow scientists to keep any Ebola virus produced from my samples in a secure laboratory for future study

I would prefer that the Ebola virus produced from my samples is destroyed after this study

**I have received the foregoing information, and it has been read to me. I have had the opportunity to ask questions about it and any questions that I have were answered to my satisfaction. I consent voluntarily to participate as a participant in this research.**

**Print Name of Participant:**

**Signature/Thumbprint/Mark of Participant:**

**Date:**

**DD/MM/YYYY**

**If illiterate**

*Participants who are illiterate should include their thumb-print or mark, and a witness who is literate should also sign (if possible, this person should be selected by the participant).*

**I have witnessed the accurate reading of the consent form to the potential participant, and the individual has had the opportunity to ask questions. I confirm that the individual has given consent freely.**

**Print name of Witness:**

**Signature of Witness:**

**Date:**

**DD/MM/YYYY**

**Statement by the researcher/person taking consent**

**I have accurately read out the information sheet to the potential participant, confirm that the participant was given the opportunity to ask questions about the study, and all questions asked by the participant have been answered correctly and to the best of my ability. I confirm that the individual has not been coerced into giving consent, and the consent has been given freely and voluntarily. A copy of this form has been provided to the participant.**

**Print name of Researcher:**

**Signature of Researcher:**

**Date:**

**DD/MM/YYYY**

**Appendix 1b Informed Consent Form, HIV testing and counseling**


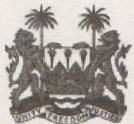

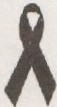

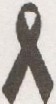


**HIV/AIDS Response Group/Ministry of Health and Sanitation**

**HCT CONSENT FORM**

**CONFIDENTIALITY/CONSENT FOR BLOOD TEST FOR HIV-** "We will take some blood from your hand. it is your choice whether or not to allow us to take the blood. We want to be sure that you understand that absolute confidentiality is maintained and your result will be given back to you. No charges are requested. The test result will just have codes (number) but no names. We would be grateful if you would let us test your blood for HIV.

!......................................................................agree to have my blood collected by finger prick and tested

for HIV,and l understand that Confidentiality is maintained and l will betold the result.

Name of Client:..................................................................................... Name of Counsellor:......................................................... ................... Date:...................................... ......... .........

Signature:.. ........................... Signature:..............................
