# Supplementary material for: Persistence of Ebola virus in semen among Ebola virus disease survivors in Sierra Leone: A cohort study of frequency, duration, and risk factors
Source: PLoS Med. 2021 Feb 10;18(2):e1003273. doi: 10.1371/journal.pmed.1003273 (PMC7875361; doi:10.1371/journal.pmed.1003273)

# Study of Persistence of Ebola virus in semen of Ebola virus disease survivors in Sierra Leone

*Special Programme of Research, Development and Research Training in Human Reproduction*

*Department of Reproductive Health and Research*

*World Health Organization*

**STATISTICAL ANALYSIS PLAN**

**COMBINED PILOT+ MAIN PHASES**

DRAFT Version 2.0

01 October 2017

**Table 1. Version Control of the Statistical Analysis Plan**

| Version | Version Date | Important Changes from Previous Version |
|---|---|---|
| 1.0 | 03 February 2017 | Initial Version –Combined Pilot-Main EBOV persistence in semen paper. |
| 2.0 | 01 October 2017 | Protocol Violations listed.<br>New health problems reported to include events reported upto 24 mo post ETU discharge. |

## I. Study description.

This is a study that aims to assess persistence of EBOV in semen among a small cohort of male Ebola survivors, in Sierra Leone, that includes assessing the feasibility of the data collection of semen. It is a descriptive observational, bio-behavioral cohort study with no random allocation applied.

**Primary objective:**

1. assess persistence of EBOV in semen among a small cohort of male survivors.

**The specific objectives:**

1. evaluate the duration and rate of persistence of EBOV in semen among male survivors.
2. determine correlates (predictors or modifiers) of the rate of persistence of EBOV in semen among male survivors.

## II. Primary Outcome

Primary outcome of interest is time from ETU discharge to confirmed negativity for Ebola. The event of interest, confirmed EBOV negativity is marked by the presence of two consecutive negative results for rt-PCR result.

## III. Study design.

This is a descriptive observational, bio-behavioral cohort study; with no random allocation applied.

Entry in the study will begin with informed consent followed by a standardized questionnaire and semen collection. All survivors with a positive RT-PCR were asked to return for a follow-up visit including repeat semen collection, after two weeks. If the second semen specimen also is positive, follow-up with repeat semen collection will thereafter take place every two weeks until the RT-PCR has been negative twice. Thereafter study participation is discontinued. The follow-up appointments will follow the same clinic flow and testing algorithms. If the initial two tests are negative, participation is discontinued. The testing interval of two weeks may be extended to 4 weeks pending on preliminary results.

Laboratory results will be shared with the participant and transmission prevention counseling and condoms provided. HIV testing and counseling will also be provided in accordance with national guidelines.

## IV. Study population

This study population consists of a convenience sample of a total of 220 male EVD survivors recruited at different time points since EVD onset recruited through survivors' networks. Of these 100 survivors were recruited during the Pilot phase of the study with enrolment occurring between 27 May to 7 July 2015, and further 120 survivors recruited between 11 November 2015 to 12 May 2016. All study participants who have provided semen samples will be included in the analyses. If follow-up specimen are missing, and if the only one available first sample is negative for EBOV, the participant will be excluded from EBOV persistence esrimation. However, if the single sample available is non-negative (i.e positive or indeterminate) for

EBOV then the participant will be included but as a censored observation. If both baseline and follow-up values from semen are missing, the participant may be included only for baseline socio-demographic characteristics, family and health history analyses. (questionnaire 1).

## V. Eligibility criteria

i. Adult Male EVD survivors
ii. Age >= 18 years
iii. In hold of a survivors certificate
iv. Two consecutive blood-negative test for EBOV

## VI. Protocol violations.

- 4 Participants never infected with Ebola discovered after recruitment.

## VII. Sample size

A sample size of 220 male participants will be used..

## VIII. Study Follow-up

Participants will be followed from baseline Visit 1, until when they have provided two consecutive negative RT-PCR semen specimens within a 2 – 4 week interval; premature discontinuation for any other reason; or up to the end of the study.

## IX. Study Site

The study conducted at two study sites, the 34 Military (M34) Hospital and Lungi study site, both in Freetown, Sierra Leone.

## X. Study Laboratories

Two laboratories responsible for sample collection and testing

i. CDC-Atlanta laboratory
ii. China-CDC laboratory

## XI. Nature of semen samples collected and test results stored database :

***Pilot Phase:***

i. Bo fresh semen samples results , collected from the study initiation to October, 2015.
ii. China-CDC fresh semen sample results – collected from October 2015 until the end of Pilot phase.
iii. Bo (CDC-Atlanta) re-read sample results
iv. China (CDC-Atlanta) retested sample results

***Main Phase*:**

i. China-CDC fresh semen sample results – collected from November 2015 to the end of Main study phase.

The primary event of interest,  will be based on the final interpretation of the PCR assays by the China CDC laboratory and  the Atlanta CDC adjusted assays (obtained by re-reading of the results from the US-CDC Bo laboratory, by the same person in the Atlanta CDC laboratory). The China CDC assay results will be considered as final interpretation values, whenever these data existed, and when they did not exist, the Atlanta CDC adjusted interpretation values will be otherwise used.

The qRT-PCR result will be handled as a dichotomous variable, either negative or non-negative. The qRT-PCR result is considered “negative” if in the final interpretation no Ebola is detected, and “non-negative” if Ebola is detected or if the result is indeterminate. In the case of no interpretation the qRT-PCR result is considered missing.  If follow-up specimen are missing, and if the only available first sample is negative for EBOV, the participant will be excluded from EBOV persistence estimation.

## XII. Data collection and procedures

Data was collected at baseline (Visit 1) with follow-up with provision of results after two weeks (Visit 2). All men offered additional (survivors', HIV) care as needed. Baseline data collected  at first visit included structured interview covering socio-demography, co-morbidity, sexual behavior, EVD course and sequelae HIV testing.  semen specimen for testing by real-time  RT-PCR, using the target gene sequences of NP and VP40 at the US CDC laboratory. All samples were stored and transported for intended analyses of virus isolation in Atlanta, US.

## XIII. Rationale for pooling the Pilot and Main study laboratory samples:

The duration between discharge from ETU to the enrolment visit for Ebola Pilot and Main phase survivors varied, with over 50% of the Main phase survivors contributing the first semen samples (at recruitment) more than one (1) year after being discharged from ETU, compared to six (6) months for the Pilot phase participants. Hence majority of the participants from the Main study will have become confirmed negative for Ebola before being enrolled (left censored), and with fewer of the participants being EBOV positive at recruitment. The data on EBOV negativity and positivity in semen for Main phase study sample still provides valuable information to strengthen the generalizability and increase the robustness of the estimates of semen persistence estimation. It was decided to pool the data from both phases, based on the recommendations from the Independent Data Monitoring Committee (IDMC) members during the meeting in November 2016.

## XIV. Calibration:

Calibration methods was proposed for exploration by the IDMC because of involvement of two different laboratories and the possible differences in the interpretation of the lab assays. The calibration and other laboratory methods comparison methodologies will be explored (Jensen AL et al, 2006; Gail MH et al, 2016) , on their feasibility for use, especially after confirmation by the laboratory experts on the validity of comparison the two target genes used in the interpretation of the EBOV, which are the VP40 and NP used by the CDC-Atlanta vs FAM and VIC used by the China CDC laboratory. Other sensitivity analyses that involve comparing EBOV persistence rates, by means of comparing the final interpretations using Atlanta-CDC re-tested China CDC samples vs fresh China CDC samples will be explored. This will be explored, if feasible.

## XV. Study Participant flow

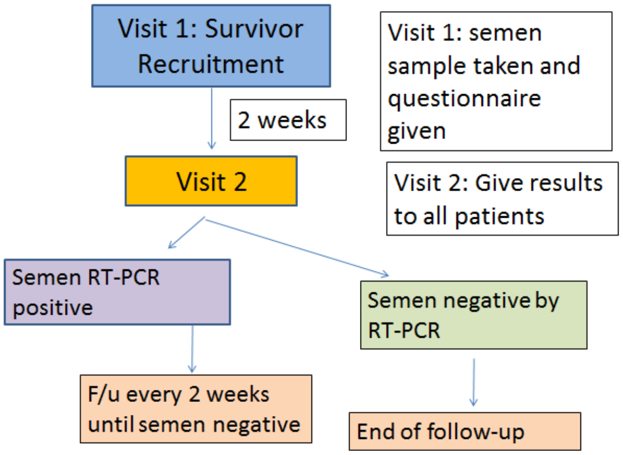


## XVI. Distribution of different laboratory samples , by lab and phase (Status as of 13 October 2016)

- **Pilot phase**

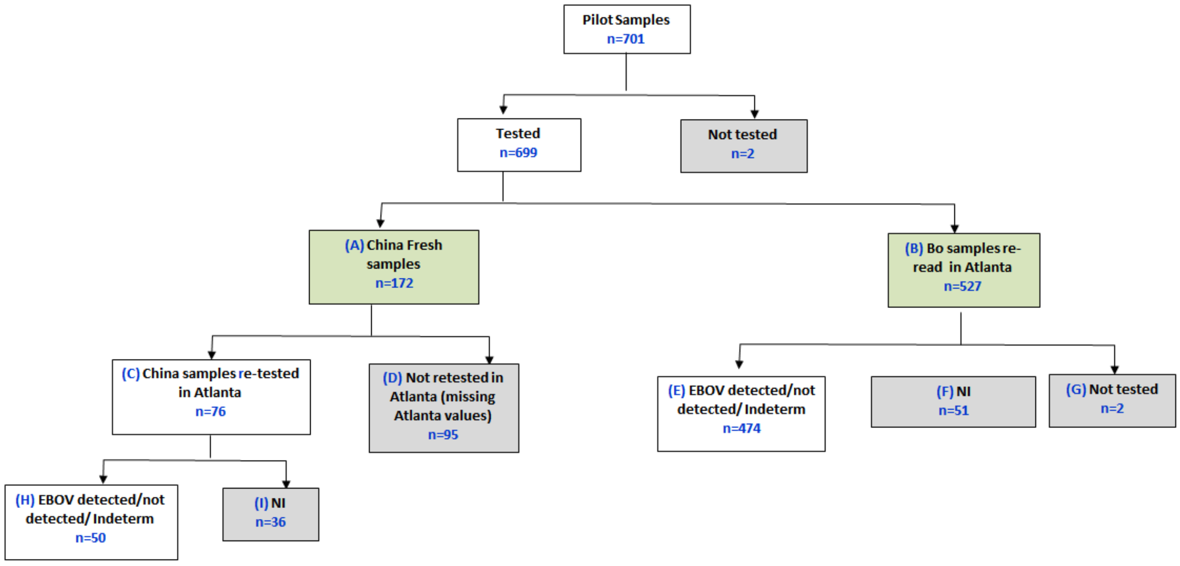


**(i) Atlanta (China re-tested+Bo re-read) samples (OLD):** B+C =525+76=>n= 601, after excluding NI => E+H=50+474=**524 samples**)

**(ii) Atlanta (Bo re-read)+ China Fresh sample (CURRENT):** A+B=174+525 =>n= 699, after excluding NI/Not tested => A-20+E=172-20+474=**626 samples**

- **Main Phase**

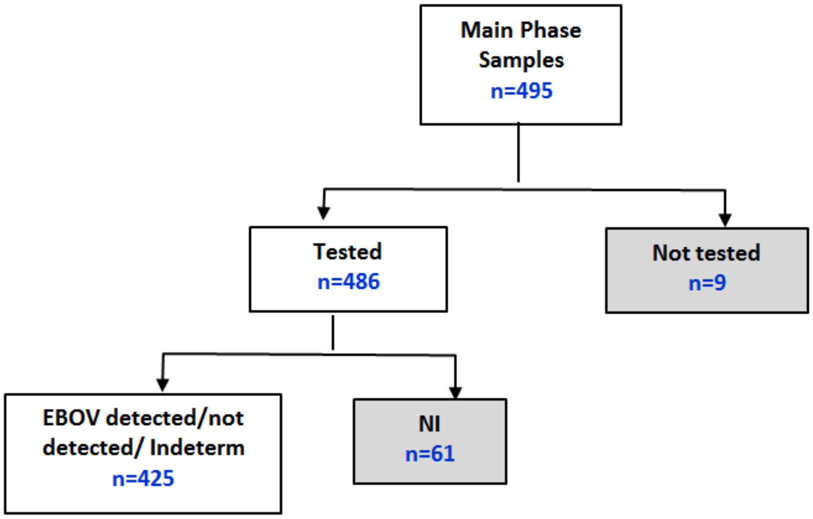

Main Phase Samples
n=495
Tested
n=486
Not tested
n=9
EBOV detected/not detected/ Indeterm
n=425
NI
n=61

## XVII. Statistical Methods.

- Study participant flowchart will be shown, that will include numbers  recruited, by phase, confirmed negative for EBOV ,well as numbers completed study, prematurely withdrawn, lost to follow-up, numbers analyzed and numbers with no semen specimen collection.
- The baseline characteristics for the entire sample recruited in both the pilot and the main study that includes new health problems reported during 24 months post-ETU discharge will be described, using corresponding data available in both phases at baseline and/or during follow-up.
- Frequencies and percentages will be reported for categorical variables. For quantitative normally distributed variables, the number of subjects, means and standard errors will be provided, while medians, interquartile range, minima and maxima will be reported for skewed non-normal quantitative variables.
- The analysis for the study outcomes, will primarily be based on every subject recruited in the study and confirmed infected with Ebola..
- Sensitivity analyses for the study outcomes that includes the left censored observations, using different time points for turning negative for EBOV, between time of discharge at ETU and time of pilot study baseline Visit 1, will be performed.
- The non-parametrics Kaplan-Meier (K-M) interval censored(IC) survival analysis, will be used to analyze time to confirmed EBOV-negative  result.(Changbin Guo et al. Paper SAS279-2014).Interval censored K-M survival curves will be plotted and the interval-sensored Proportional Hazards model will be used to adjust for the  potential confounders/independent variables. Interactions between covariates in the model will be studied and if significant, included in the final model. For other time-to-event outcomes, right censored techniques survival techniques will be used (Changbin Guo et al(2014), Lee T.1992). Parametric Weibull survival models will also be explored.
- RT-PCR results will be used to define the event in the survival analysis, and will be complemented by the virus isolation results as well as ct-values of RT-PCR analyses. Virus isolation results may be used for a sensitivity analyses in addition to a descriptive reporting of results in concjunction with RT-PCR -results.
- For the categorical (binary) repeated outcome on Uptake of advice to prevent spread of ebola, the log-binomial generalized estimating equations (GEE) regression models will be used to  will be used to estimate the risk, risk ratios, which will account for the exposure duration, as well as correlated responses within subjects, over time. The potential confounders/independent variables will be adjusted for in the model (Molenberghs and Verbeke 2005). Because the duration has censoring, the GEE model might need to be adjusted so that the censoring mechanism could be factored into inference due to unexpected attrition, dropout and/or incomplete response. (SAS/STAT® 13.2 User's Guide GEE procedure (experimental).
- Two-sided tests and 5% significance levels will be used, and 95% confidence intervals for all relevant parameters.  SAS statistical packages will be used for the statistical analyses.
- Open-ended questions will be listed and coded for meaningful comparisons of their distribution.

## XVIII. Tables and Figures. See ANNEX 2.

**ANNEX 1. Description of methods and variables to be used for each of the objectives of the study.**

## I. BASELINE DISTRIBUTION

- **Study population:** Everyone recruited in the Pilot and Main phases
- **Socio-demographic and socio-economic variables:**
  - Age
  - Education
  - Marital status
  - Type of relationship to household members
  - Number of household members, including self
- **Ebola history variables:**
  - Vomiting during ebola? (Y/N)
  - Diarrhea during ebola? (Y/N)
  - Too sick to relieve self on toilet during ebola? (Y/N)
  - Too sick to drink for ≥1 day during ebola? (Y/N)
  - Ebola severity score variable (ranked) based on 4 indicators: Vomitting, Diarhoea, Too sick to relieve self on toilet and too sick to drink >1 day during Ebola as ranked follows:
    - 0-None of the 4 symptoms
    - 1-Any of the 4 symptoms
    - 2-Any 2 of the 4 symptoms
    - 3-Any 3 of the four symptoms
    - 4-All the four symptoms
    - at least 1, 2, 3 or more symptoms
  - Number of household members with Ebola in household

- **Post-ebola current health variables, reported 24 months after ETU discharge:**
    - Overall health and well-being compared to when had ebola
    - Any health problems, post-Ebola recovery? (Y/N)
        - New health problems (specify): Eye/Vision problems? (Y/N)
        - New health problems (specify): Joint pains? (Y/N)
        - New health problems (specify): Psychological problems including enxiety and depression? (Y/N)
        - New health problems (specify): Sexual problems? (Y/N) –
    - Ever tested for HIV? (Y/N/DK)
- **Post-ebola sexual activity variables:**
    - Resumed sexual activity?
    - Engaged in sexual activity ≤ 3months after Ebola recovery? (Y/N)
    - How often used condoms ≤ 3months after Ebola recovery?
    - Sexual desire? (Y/N)
    - Difficulty erection or ejaculation? (Y/N)

❖ **Post-ebola information variables:**
- Advice on resuming sex, post-Ebola
- Advice on Condom use, post-Ebola

❖ **Statistical Methods:**

i) Number of participants, number of missing values, frequencies and percentages will be reported for categorical variables.

**ii)** Number of participants, number of missing values, minima, maxima, means and standard deviations (SD), median (interquartile range (IR)) will be reported.

**SPECIFIC OBJECTIVES**

1. **Evaluate the duration and rate of persistence of EBOV in semen, at key time points, among confirmed blood-negative survivors, counting from symptom onset (or rt-PCR negative in blood) and with persistence assessed by rt-PCR as well as virus isolation (sensitivity analyses, correlation or descriptive reporting)**

   - **Study population:** All male Pilot and Main phase participants (out of the 220 enrolled) confirmed as infected with Ebola and having at least 2 valid semen sample results.

***The rate of persistence of Ebola in semen will be evaluated using interval-censored techniques as follows:***

The participant intervals for the interval censored methods will be considered as:

- *left censored*; in case of 2 consecutive negative EBOV test in semen at the first two visits following recruitment. The left-censored interval will count from ETU discharge date (which is the origin, or Time 0) to date of the first visit (Visit 1);

- *Right-censored;* This occurs in absence of two consecutive negative EBOV results at any time during follow-up from recruitment. The right-censored interval will count from the last test result date (last available visit) to infinity;

- *Interval censored*;

  in case the event of interest (confirmed negative result for EBOV in semen) occurs between two dates during follow-up. The interval will be between the latest date of either *a positive or indeterminate result* and the date of *first of two consecutive negative tests*.

- ***Statistical Methodology***

  EBOV persistence rate in semen is defined as the first time within the entire duration observed from the start of Ebola symptoms (or alternatively, confirmed blood negative for EBOV), that two consecutive EBOV semen results show negativity.

  Overall cumulative EBOV persistence rate will be estimated using the interval censored K-M techniques, using SAS Procedure ICLIFETEST(Changbin Guo et al (2014).. The 95% confidence intervals based on the complementary log-log transformation will be reported. The interval-censored PH model that will adjust for potential confounders, and interactions, if important, will be fitted and estimates of relative risk (95% CI) while controlling for other confounders will be obtained using SAS Procedure ICPHREG and using piecewise constant baseline hazard (SAS Institute Inc 2014).

2. **Determine correlates (predictors or modifiers) of the rate of persistence of EBOV in semen among male survivors.**

In addition to the time-to-event and censoring status variables (Survival analysis) for the endpoint as indicated in Specific Objective (1), the baseline factors will be taken into consideration for adjustment for, in the analysis using the interval censored PH model.

- **Study Population:** Main and Pilot phase participants.
- **Potential Confounders** (IC PH model)

  Baseline variables listed on Page 10-11 -

**Methods:**

1. Univariate IC PH regression with persistence in semen as dependent variable, and each independent variable or potential confounder as independent.
2. Multivariate stepwise IC PH regression where the factors will be fitted, one at a time in a univariate IC PH model
3. The values of -2Log Likelihood (-2LogL) between the univariate model and the null (baseline hazard) model will be compared to determine which variables significantly reduce the value of -2LogL.
4. All factors in (3) which are important on their own (p-value of <=0.15) will be fitted together in a full model.
5. Only the factors that when omitted from the model in (4) lead to significant increase in -2LogL (p<0.15) are retained in the model.
6. Finally variables not important on their own (in 4) will be added into the model to determine whether they can be important in the presence of other. This is done until when no factor added or omitted from the model does not significally change the value of -2LogL
7. Each factors will be omitted from the full model one at a time, to determine its importance in the model, and all factors with p-value>0.15 will be dropped from the model
8. Hazard ratio (HR) between different levels of the important independent variables (univariate and in the final multivariate models) will be estimated.
9. Interaction effect between levels of the important variables will also be assessed.

## References


1) Molenberghs and Verbeke. Models for Discrete Longitudinal Data.2005.
2) Lee ET. Statistical methods for survival data analysis. 2nd ed. New York: Wiley and Sons, 1992.
3) SAS/STAT® 13.2 User's Guide GEE procedure (experimental).Weighted Generalized Estimating Equations under the MAR Assumption. http://support.sas.com/documentation/cdl/en/statug/67523/HTML/default/viewer.htm#statug_gee_details06.htm
4) SAS Institute Inc. 2014. SAS/STAT® 13.2 User's Guide.Cary, NC: SAS Institute Inc.
5) Deen GF et al.NEJM 2015.Ebola RNA Persistence in Semen of Ebola Virus Disease Survivors — Preliminary Report
6) Changbin Guo et al. Paper SAS279-2014. Analyzing Interval-Censored Data with the ICLIFETEST ProcedurePetersen PH, Ricos C, Stockl D, et al.
7) Asger Lundorff Jensen, Mads Kjelgaard-Hansen. Method comparison in the clinical laboratory. Invited Review. Veterinary Clinical Pathology. 2006;35(3):276-286.
8) Collett D. Modelling Survival Data in Medical Research. London: Chapman & Hall; 1994.
9) Gail MH et al. Calibration and seasonal adjustment for matched case-control studies of vitamin D and cancer. *Stat Med 2016 Jun 5;35(13):2133-48. Epub 2016 Jan 5.*

**ANNEX 2. Tables and Figures.**

| Table or Figure | Description |
|---|---|
| **Figure 1.1** | Study subjects disposition flowchart, by Phase and Overall |
| **Table 1.1** | Male participants' distribution of selected baseline characteristics, by Phase, Overall |
| **Table 2.1** | Interval censored Kaplan-Meier estimates of the rate of Persistence of Ebola in semen |
| **Figure 2.1** | The KM Interval censoring rate of EBOV persistence in semen, with Weibull fit. |
| **Figure 2.2** | The KM Interval censoring rate of EBOV persistence in semen, by phase |
| **Table 2.2** | Univariate and Multivariable Interval censored Proportional Hazards model regression estimates of relative risk of being confirmed negative for Ebola virus in semen |

**Figure 1.** Observational cohort study subjects disposition

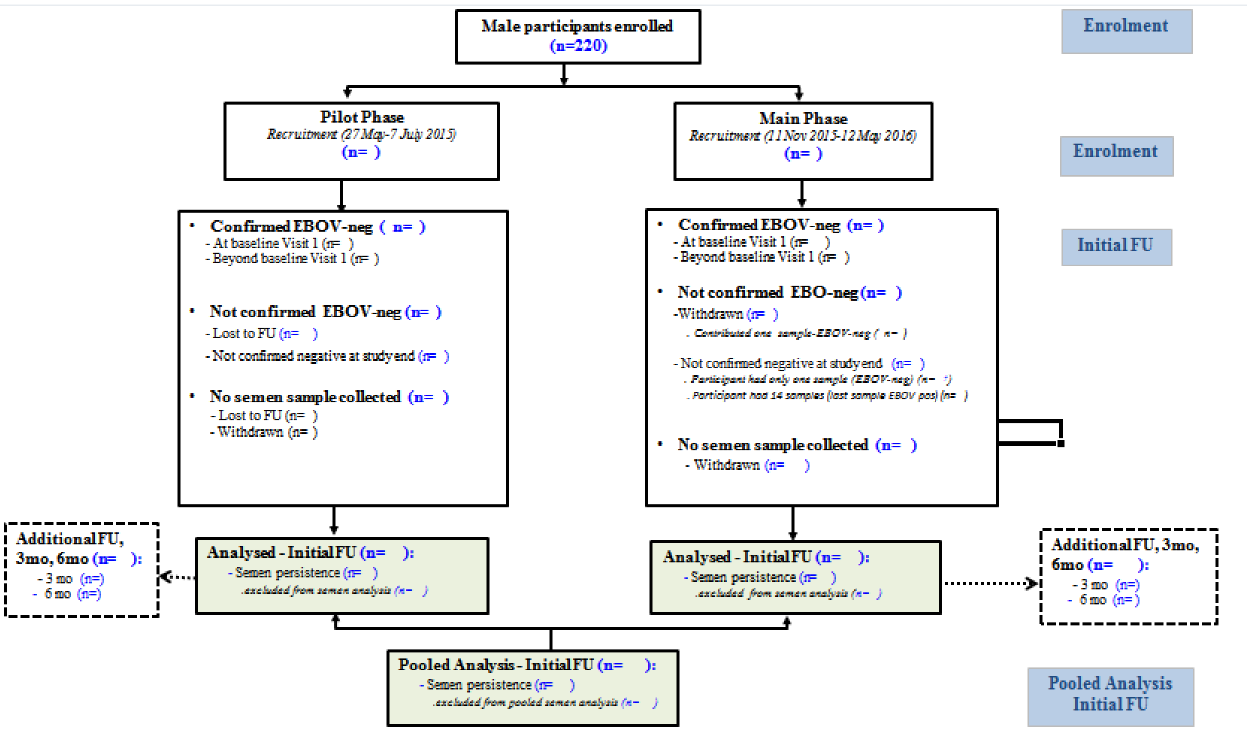

Supplement: S1 Statistical Analysis Plan — (PDF) [file pmed.1003273.s003.pdf]
