# Supplementary material for: Persistence of Ebola virus in semen among Ebola virus disease survivors in Sierra Leone: A cohort study of frequency, duration, and risk factors
Source: PLoS Med. 2021 Feb 10;18(2):e1003273. doi: 10.1371/journal.pmed.1003273 (PMC7875361; doi:10.1371/journal.pmed.1003273)
Supplement: Attachment — Submitted filename: response to reviewers April 2020.docx [file pmed.1003273.s004.docx]

**Response to Editor and reviewers**

*Dear Editor,*

*Thank you so much for providing these comments and for sharing important insights from the reviewers.*

*Please find our point-by-point answers below, together with the uploaded manuscript in track changes and clean versions, as well as indicated supplementary material.*

*We have provided our answers in italics, with citations of revised manuscript text highlighted and with page references to the revised/marked up version for easy reference.*

*We will upload the files including the figures per instructions. We notice that the addition of 95% CI in some cases make them less clear, given the different stratifications. Please let us know if you prefer us to present these differently.*

**Requests from the editors:**

Title-Please revise your title according to PLOS Medicine's style. Your title must be nondeclarative and not a question. It should begin with main concept if possible. "Effect of" should be used only if causality can be inferred, i.e., for an RCT. Please place the study design ("A randomized controlled trial," "A retrospective study," "A modelling study," etc.) in the subtitle (ie, after a colon).

*The title has been edited.*

Persistence of Ebola virus in semen among survivors in Sierra Leone: A cohort study of frequency, duration and risk factors

Abstract
Please structure your abstract using the PLOS Medicine headings (Background, Methods and Findings, Conclusions).

Background- Provide the context of why the study is important. The final sentence should clearly state the study question.

*The abstract has been overall revised inclusive of above instructions. (pages 3-4)*

Please ensure that all numbers presented in the abstract are present and identical to numbers presented in the main manuscript text.

*The numbers have been updated (months reported in days, and the figures reported to one decimal place, as per table 2) (page 3)*

* Please include the study design, population and setting, number of participants, years during which the study took place, length of follow up, and main outcome measures. * Please quantify the main results (with 95% CIs and p values).

*This has been revised, and we have included 95%CI and p-values (page 3)*

* Please include the important dependent variables that are adjusted for in the analyses.

*These are included. (page 3)*

* Please include the actual amounts and/or absolute risk(s) of relevant outcomes (including NNT or NNH where appropriate), not just relative risks or correlation coefficients. (example for absolute risks: PMID: 28399126).

*For our study, the EBOV confirmed negativity is the outcome of interest, which is a time-to-event, and we estimate the Hazard rate (HR) per different independent co-variates/sub-groups and compare these groups through the Hazard rate ratios (HRRs) estimates. In our study we did not analyze the effects of a treatment, related to the outcome, so the NNT / NNH would from that perspective be less relevant. We welcome any comments on this and are happy to adjust further as needed.*

* Please include a summary of adverse events if these were assessed in the study. *NA*

* In the last sentence of the Abstract Methods and Findings section, please describe the main limitation(s) of the study's methodology/study design.

*This has been added. (page 4)*

Abstract Conclusions:
* Please address the study implications without overreaching what can be concluded from the data; the phrase "In this study, we observed ..." may be useful.
* Please interpret the study based on the results presented in the abstract, emphasizing what is new without overstating your conclusions.
* Please avoid vague statements such as "these results have major implications for policy/clinical care". Mention only specific implications substantiated by the results.
* Please avoid assertions of primacy ("We report for the first time....")

*Thank you, the abstract has been overall revised per above instructions. (page 4)*

The Data Availability Statement (DAS) requires revision. For each data source used in your study:

a) If the data are freely or publicly available, note this and state the location of the data: within the paper, in Supporting Information files, or in a public repository (include the DOI or accession number).

b) If the data are owned by a third party but freely available upon request, please note this and state the owner of the data set and contact information for data requests (web or email address). Note that a study author cannot be the contact person for the data.

c) If the data are not freely available, please describe briefly the ethical, legal, or contractual restriction that prevents you from sharing it. Please also include an appropriate contact (web or email address) for inquiries (again, this cannot be a study author).

*The following section has been added under a separate heading in methods, and relates to all the data in the study:*

*Contractual agreements between the study parties (HRP/WHO, US CDC, China CDC, and the MoH SL) exists and data rights reside with MOH-SL, the material owner. Inquiries related to the data, may be directed to Study Data Oversight Committee (*[*SIS@who.int*](mailto:SIS@who.int)*) with Subject:  “Inquiry on Ebola Semen persistence study data”.*

Please use Vancouver style for references and place all references within square brackets

*The style has been revised to Vancouver with brackets.*

Author summary

At this stage, we ask that you include a short, non-technical Author Summary of your research to make findings accessible to a wide audience that includes both scientists and non-scientists. The Author Summary should immediately follow the Abstract in your revised manuscript. This text is subject to editorial change and should be distinct from the scientific abstract. Please see our author guidelines for more information: <https://journals.plos.org/plosmedicine/s/revising-your-manuscript#loc-author-summary>

*Thank you, this has been added, (highlighted in yellow in the revised version inclusive of tracked changes), pages 5-6.*

Introduction

Please address past research and explain the need for and potential importance of your study. Indicate whether your study is novel and how you determined that. If there has been a systematic review of the evidence related to your study (or you have conducted one), please refer to and reference that review and indicate whether it supports the need for your study.

Please conclude the Introduction with a clear description of the study question or hypothesis.

*This has been revised, inclusive of presenting the systematic review done by us prior to study start. (pages 7-8, introduction)*

Methods

Please provide details or citations of all interview guides used in the study. If these are not published, please provide a copy as SI files.

*These guides are provided as SI files.*

Please report the number of [patients, samples, etc] and dates of recruitment, and account for all methods used in your study.

*This has been presented and revised further for clarity. (study design: pages 8-9)*

Ethics approval- please provide details and name of the approving committee in the methods

*This is included under sub-heading Ethics in Methods, (highlighted in yellow in the revised version).*

*Ethical permission was granted from the Sierra Leone Ethics and Scientific Review Committee* *and the WHO Ethical Review Committee (No. RPC736).*

Please ensure that the study is reported according to the STROBE guideline, and include the completed STROBE checklist as Supporting Information. Please add the following statement, or similar, to the Methods: "This study is reported as per the Strengthening the Reporting of Observational Studies in Epidemiology (STROBE) guideline (S1 Checklist)."

The STROBE guideline can be found here: <http://www.equator-network.org/reporting-guidelines/strobe/>

When completing the checklist, please use section and paragraph numbers, rather than page numbers.

*The reporting has been revised to follow STROBE and the checklist has been included, and the statement added. (STROBE added as an SI)*

Did your study have a prospective protocol or analysis plan? Please state this (either way) early in the Methods section.

a) If a prospective analysis plan (from your funding proposal, IRB or other ethics committee submission, study protocol, or other planning document written before analyzing the data) was used in designing the study, please include the relevant prospectively written document with your revised manuscript as a Supporting Information file to be published alongside your study, and cite it in the Methods section. A legend for this file should be included at the end of your manuscript.

*The statistical plan of analysis is shared as supplementary information*

b) If no such document exists, please make sure that the Methods section transparently describes when analyses were planned, and when/why any data-driven changes to analyses took place.

*NA*

c) In either case, changes in the analysis-- including those made in response to peer review comments-- should be identified as such in the Methods section of the paper, with rationale. Please provide references to databases used in the study

*The Sierra Leone Ebola persistence database used was as of status dated 03-April-2017. This has been added to methods. No changes were made on the statistical methods based on peer review.*

Please provide further details about the housekeeping gene used in this study, along with the sequence as supplementary information

*This following has been added to methods. Please let us know if we can supply more in case assessed to be needed.*

A housekeeping gene sequence assessed specimens’ quality including the presence of cellular beta 2 microglobulin (B2M) mRNA (Invitrogen), that served as a RNA extraction and sample authenticity control.

Page 6 footnote-please move this to main text

*This has been moved.*

Is it necessary to mention FAM and VIC reporter dyes on page 7 without any background? Please revise so that the methods can be read and understood by a general audience

*This has been revised and the language edited to fit a general audience. (outcome variable: page 10)*

Please provide details about when this study was carried out, when were samples collected and details of follow-up.

*This section in methods has been revised and added to, and we also refer to our baseline publication, Deen et al (2017) in NEJM as well as our papers presenting the implementation (no results) of the study and specifically the counselling process. (Results 1^st^ para; pages 13-14)*

PLOS does not permit "data not shown.” Please remove this claim, or do one of the following: a) If you are the owner of the data relevant to this claim, please provide the data in accordance with the PLOS data policy, and update your Data Availability Statement as needed. b) If the data not shown refer to a study from another group that has not been published, please cite personal communication in your manuscript text (it should not be included in the reference section). Please provide the name of the individual, the affiliation, and date of communication. The individual must provide PLOS Medicine written permission to be named for this purpose. c) For any other circumstance, please contact me ASAP.

*This has been revised, a longer presentation of the comparative data has been presented and inserted in the text, and the data availability statement has been inserted, per the comment to DAS above. (page 13)*

Discussion

Please present and organize the Discussion as follows: a short, clear summary of the article's findings; what the study adds to existing research and where and why the results may differ from previous research; strengths and limitations of the study; implications and next steps for research, clinical practice, and/or public policy; one-paragraph conclusion.

*The discussion has been revised. (pages 18-23; inclusive of a revised conclusion)*

**Comments from the reviewers:**

**Reviewer #1:**

*Thank you so much for careful and valuable comments!*

In the proposed paper, the authors present the follow-up of semen of Ebola survivors. They find that Ebola virus was present in many individuals, even a year after follow-up. This presents an interesting follow-up to the original paper (Deen et al., NEJM) as it includes some longitudinal data not included in the original paper. Nevertheless I was confused by the statistical approach taken.

*We used the semi-parametric interval-censored proportional hazards (IC PH) multivariable model (not a Weibull model) for estimation of the risk factors. The parametric Weibull fit was super-imposed to the non-parametric Kaplan Meier's IC survival curve to acertain how best it fitted the data (Figure 1) .*Collett D. Modelling Survival Data in Medical Research. London: Chapman & Hall; 1994.*

*We have now included the brief description and reference(*) to the strategy that we applied for multivariable model building. (Survival analysis: Page 12)*

My major concern/confusion is with the main regression analysis. The authors conduct a multivariable Weibull regression, where they include an interaction term between education and living with extended family. They also dedicate an entire table (Table 3.2) to stratified results for living and not living with extended family. The rationale for this approach was not set out in the introduction or methods and only briefly mentioned in the results. I also struggle to come up with a mechanistic rationale as to why this would be of particular interest. In the discussion there is a mention of reinfection risk over time, but then again, I struggle with why living with extended family is a good marker of this. I think this focus is an unfortunate distraction for the paper and I would consider removing it.

*The interaction effects of living with or without extended family, was identified in the analytical work when looking at socio-demographic associations, with the purpose to generate hypotheses. We agree this may deviate from the main message and have revised to remove it per the above suggestion.*

The 95% confidence intervals for the Kaplan-Meier curve (Figure 1) look very narrow, especially as there are only 220 individuals. The authors should clarify how these were calculated. The authors should also include 95% confidence intervals for the Weibull fit in Figure 1.

*We have included the 95% CI for the Weibull fit for Figure 1.*

*The standard errors (SE) estimates for the IC Kaplan Meier survival estimates is based on the multiple imputations (in this cases 1000 imputations) that gave rise to the standard errors of the survival estimates reported. The algorithm used to estimate the SE is efficient Expectation-Maximization Iterative Convex Minorant (EM-ICM) algorithm (ref Wellner JA, Zhan Y. A hybrid algorithm for computation of the non-parametric maximum likelihood estimator from censored data. Journal of the American Statistical Association 1997;92:945-59.).*

There are no uncertainty intervals presented in Figure 2 and Figure 3 - these need to be included.

*The 95% CIs have now been added*

The authors refer to 'data not shown' to say their sample is representative of the wider population. There is sufficient space in the article to set out the comparisons between their cohort and the wider population in a table - and show how the two populations are different and the same.

*This section has been revised and more information added. (Representativity of sample; page 16)*

The authors use two sequential negative tests as a marker of being a true negative. It would be useful to set out how frequently a negative was followed up by a positive test to give readers an idea of how robust this definition is.

*Thank you, the following details have been added:*

37 of 88 had at least once prior to the end point, one negative test immediately followed by a positive one. Of the 7 who were right censored, 4 of them had a single negative result which was immediately followed by a positive results (also this occurring at least once)

Lower CT score is used as a proxy for severe disease. The rationale for this proxy association should be referenced (e.g., Faye et al., PLoS Medicine). Viral load itself is of interest (irrespective if it is linked to symptoms), however, it will depend on when it was tested during acute illness - this weakness should be discussed.

*Thank you, this has been revised and inserted, (this reference had been mistakenly omitted). The weakness of timing of the acute value is discussed and has been expanded on in the study limitations as follows:*

The acute Ct-values in our study were not available for all participants, were taken at different points in time during acute disease and were analyzed by different laboratories, which may have diluted the associations observed. The lowest available acute Ct-value was consequently retrieved to mitigate mis-classification.

On page 6, I do not understand the sentence 'A housekeeping gene sequence assessed specimens' quality through compatibility with human DNA' - the authors should clarify exactly what was done.

*Thank you, this paragraph has been revised to include more detailed information (please see also above under Editor’s comments)*

The authors use a simple dichotomization of greater/less that a CT of 27 for high/low disease severity - it would be useful to look at additional bins of CT (or use a spline) to see the full relationship between the two.

*We opted for a dichotomized approach, nd did also analyze symptoms during acute disease. Given the fact that the acute CT-values were analyzed by different laboratories and taken at different points in time over the course of acute disease, and guided by their distribution, we opted for this dichotomized approach around the mean. The limitations of data have been further explained in the text.*

**Reviewer #2:**

In this manuscript, Thorson et al analyzed the persistence of EBOV RNA in semen from a longitudinal cohort of survivors in Sierra Leone. Using two different EBOV RT-PCR, they monitored the kinetics of EBOV persistence in semen and established the survival function of EBOV RNA persistence over time according to time after discharge and to initial viremia and age. They demonstrated that the persistence correlated with age and other factors such as the occurrence of diarrhea during acute disease. The study is well designed and managed, the statistical analyses are robust, and the main strength of the study is the longitudinal follow-up in survivors with samples obtained very frequently. Some results obtained with this cohort have previously been published in NEJM by the authors and the current report provides the complete and final follow-up of the cohort.

*Thank you so much for these comments! Like noted, the baseline characteristics of the cohort was published earlier in NEJM, and in this analysis we present the longitudinal analysis, allowing for estimates of rates of persistence over time as well as of a population mean persistence.*

The main weakness of this study is the lack of real novelty of the results, as a lot of reports describing the persistence of EBOV RNA in semen have already been published, with quite comparable conclusions. For example, the most recent publication (Keita, Open Forum Infect Dis), not cited by the authors, described a similar cohort with analysis of several body fluids including semen. Compared to the previous reports, no new discovery was provided in the current study.

*Thank you so much for these comments.*

*Other studies (including Keita) have presented or modeled long duration of persistence in semen, in single cases or in small cohort studies, with limited or irregular follow-up. These have reported highly variable results and like concluded in the review by Schindell et al., information on rates and population mean values of duration of persistence over time are needed to complement the hypothesis raised in these case reports and limited cohorts or cross-sectional studies. None of the earlier studies have hence presented population estimates of rates of semen persistence over time, that are based on a similarly powered sample and with regular and more frequent follow-up, applying a time -to-event/survival analysis. Keita et al’s study, provides insights to duration of persistence in semen in individual cases (n <10), followed with 6 monthly intervals, and by adding modelling. Comparably, our study includes a larger sample who were followed every other week, to allow for a more robust survival analysis, including confidence intervals. Likewise, the magnitude and rate over time of semen persistence, is in our study based on a powered sample with frequent follow-up. We present a 6 month persistence rate based on this full cohort analysis, of 75% with CI, whereas Keita presents a 6 month estimated rate at 60%, based on partly modelled data, and per their results from < 10 men. We have added a reference to the Keita study to our paper.*

*Further, our study is the first to analyze associations between sociodemographic, acute disease severity and sequelae in a multivariate analysis in a powered study. We also present sexual risk behavior after discharge data, presenting important evidence to inform counselling and semen testing programmes.*

Major concerns.

It would have been interesting and important to perform viral isolation in addition to viral RNA detection to obtain information about the risk of EBOV sexual transmission according to the time after discharge. In addition, viral isolation would have allowed to perform genome sequencing of persisting viruses. These data would have constituted an important advance in the field.

*We agree fully, and we have also stated the limitations of RT PCR versus virus isolation in the paper. Attempts for complete virus isolation were made, but did not happen as planned due to challenges with shipping and laboratory capacity in-country. We have however also added the paper by Whitmer, thank you so much for suggesting this. In fact the Whitmer paper includes data from the survivors included in this cohort (together with data form the US) and hence carries extra relevance related to insights for this study. The Whitmer paper suggests that RT PCR positive findings are likely to correlate to viral persistence rather than latent infection, and that persistence in semen may constitute a viral reservoir; including active viral replication. (Ebola virus detection: page 9)*

The authors should have cited and discussed more exhaustively the existing literature about EBOV persistence such as Whitmer 2018 (Cell Reports 22, 1159), Keita 2019 (OFID 6,12), Barnes 2017 (CID 65:1400)…

*We agree these are important papers, thank you for pointing this out! We have per above added the Whitmer and Keita (2019) papers as well as revised content to comment on these, to the revised version. (Discussion: page 17 and 21) The Barnes case report is likewise included in the review by Schindell, which is referred to in the introduction.*

Minor concerns.

In table 1, the information about the sexual desire appear twice, but with different values

*We collected symptoms and signs longitudinally during follow-up. In the first case the analysis shows if there has been any report of less sexual desire reported any time over 24 months of follow up. Then we also present sexual behavior including changes in sexual desire, immediately after recovery (up to 3 months after). We have revised the wording and order in table 1 to make this clearer.*

**Reviewer #3:**

The study by Thorson et al. addresses a public health problem for Ebola virus disease by providing a thorough, and very well written, analysis of samples and data rather difficult to achieve. I only suggest minor changes.

While the study provides academically sound results, their public health relevance could be improved by addressing some virological issues.

Results of the same study was already published earlier (Deen et al. 2017). Of 220 participants EBOV RNA was detected up to 1.5 years after ETU discharge. They stated, that "These data showed the long-term presence of Ebola virus RNA in semen and declining persistence with increasing time after ETU discharge.

*Thank you so much for these insightful comments!*

*Deen et al represents the baseline analysis of the here presented cohort, which is a cross-sectional analysis* *of the sample included, and not a longitudinal follow-up analysis (which is presented here). That paper, as well as some other case studies have presented single cases of long duration of persistence. We present here a full cohort analysis, which allows for an estimate of a population mean duration of persistence as well as estimates of population rates of persistence over time. The cross-sectional design in our first paper, suffer from biases and cannot provide information other than as a description of the sample at baseline. No inference can be drawn from that analysis and interpretations are intentionally limited in their character. In the here presented cohort we further include multivariate regression analysis for associations to risk factors of persistence as well as a presentation of sexual behavior.*

" An added value of the published data could be achieved by evaluating and concluding directly the risk of sexual transmission of EVD. Barnes et al. (CID 2017:65) describe in their case report the isolation of viable EBOV in semen up to day 32 (after clinical recovery from EVD) and active replication in cells present in semen. Unfortunately, the submitted results does not relate to viable virus in semen but only viral RNA fragments. Although this might be difficult to achieve, it would strengthen any recommendation based on the presented data. By using an internal standard the amount of virus genome copy numbers could be used instead of PCR ct-values for a moreinformative conclusion and risk analysis. It has been shown by Barnes et al. that low ct-values of EBOV can be detected in semen within the first weeks/months after recovery from EVD. It would be helpful to correlate ct-values with amounts of viable virus (either directly from the clinical samples or by spiking experiments). Further, results are only defined as positive and negative by ct-values below or above 40. Depending on the PCR protocol a more informative outcome and conclusion could be achieved by presenting detailed ct-values or even better viral genome copy numbers.

*We agree fully, and we have also stated the limitations of RT PCR versus virus isolation in the paper. Attempts for complete virus isolation were made, but did not happen as planned due to challenges with shipping and laboratory limitations in-country. We have however also added information from a paper by Whitmer, which includes data from survivors’ specimen included in this cohort and hence carries extra relevance related to insights for this study. The Whitmer (2018) paper suggests that RT PCR positive findings are likely to correlate to viral persistence rather than latent infection, and that persistence in semen may constitute a viral reservoir, showing active viral replication. (Discussion: page 21) Further the verified transmission chains of sexual transmission caused by persistence in survivors > one year after discharge, are included in the introduction.*

About the housekeeping gene target as internal control: was RNA being used after DNase treatment to avoid measuring DNA fragments? Please specify in methods section. Quality control for a RNA target should be undertaken by use of a RNA internal control.

*Thank you, we have clarified the information on the housekeeping gene to read as follows:*

*A housekeeping gene sequence assessed specimens’ quality including the presence of cellular beta 2 microglobulin (B2M) mRNA (Invitrogen), that served as a RNA extraction and sample authenticity control.*

Despite recommendations for safe sex, a third of survivors had a low uptake. Did you include testing of sex partners for monitoring of a possible transmission/infection? This could easily be tested by serology? This would further allow calculating or discussing the risk for sexual transmission and resulting infection with EBOV?

*It would have been interesting to follow sexual behaviour over time and to test partners with a longer follow-up than three months (ie also after the peak of the epidemic, for sero-conversion) for sexual transmission, but this was unfortunately not included. We do however present data on household infections related to persistence, even if the causal direction of those associations are not known.*

As the author's state in their study, a high proportion of male survivors regularly had unprotected sex after recovery from EVD, and obviously without causing another outbreak. How can this be explained?

*The survivors discharged in this study were asked for their sexual behavior over the three months post discharge, since recommendations at the time stipulated sexual risk reduction for this time period. That period, the outbreak was still ongoing and distinguishing between sexual and other transmission was difficult, even if some data on this has been presented, outside our cohort. There are further published reports of verified sexual transmission chains from persistence, > 1 year after discharge. Also, very little is known of how eventually mode of transmission could hypothetically shape disease development related to severity. We note that the current epidemic in DRC has again flared up, without a recognized clear source of transmission, and hence sexual transmission could be a hypothetical explanation.*

**Reviewer #4:**

Thorson and colleagues present a thorough analysis of the magnitude and duration of Ebola virus persistence in semen from survivors in Sierra Leone. The authors also include analysis of potential risk factors that influence persistence.

Overall, I had few, if any, concerns with the manuscript. There were a few instances of redundant use of terms in the same or back-to-back sentences; however, these could be rectified with an additional thorough proofread.

The authors should be commended on their analysis. In particular, the authors provide evidence for more thorough behavioral guidance and analysis regarding sexual/reproductive health for EVD survivors. In addition, the authors have identified correlations between age and duration of persistence. As the authors point out, this could be related to a number of biological variables that should be further studied. Taken together, the authors have provided an informative manuscript that expands on their prior work while also providing guidance for public health practices.

*Thank you so much for these encouraging comments! We have made a careful proof reading from our side to correct language.*
